# Estimated number of people infected with hepatitis B and C virus in Germany in 2013: a baseline prevalence estimate using the workbook method

**DOI:** 10.3389/fpubh.2025.1471256

**Published:** 2025-04-07

**Authors:** Katrin Kremer-Flach, Ruth Zimmermann, Matthias an der Heiden, Sandra Dudareva

**Affiliations:** ^1^Department of Infectious Disease Epidemiology, Robert Koch Institute, Berlin, Germany; ^2^European Program for Intervention Epidemiology Training (EPIET), European Centre for Disease Prevention and Control (ECDC), Stockholm, Sweden; ^3^Postgraduate Training for Applied Epidemiology (PAE), Robert Koch Institute, Berlin, Germany; ^4^Institute of Public Health, Riga Stradins University, Riga, Latvia

**Keywords:** hepatitis B, hepatitis C, people who use drugs, migrants, Germany

## Abstract

**Introduction:**

Hepatitis B (HBV) and hepatitis C (HCV) viral infections are uncommon in Germany, though these infections have a higher prevalence among certain subpopulations, such as some first-generation migrant groups, people who inject drugs (PWID), and HIV-positive men who have sex with men (HIV+MSM). Repeated estimates of the number of people infected with HBV and HCV are essential to facilitate the monitoring and elimination efforts by 2030. We estimated the total number of people infected with HBV and HCV in Germany, and the number in each specific subpopulation. We based our calculations on data from 2013, a year that we strategically chose to coincide with the availability of data from serological surveys, the advent of highly effective antiviral therapy for HCV, and significant migrant flows in the following years.

**Methods:**

We used the workbook method, a technique that combines subpopulation size and prevalence data. We included different population groups (general population excluding vulnerable groups, migrants stratified by nationality, people who inject opioids (PWIO) and HIV+MSM). We estimated the number of people infected with HBV and the number of people infected with HCV. Estimates of the number of people infected with HBV and HCV are reported with the lower and upper confidence limits.

**Results:**

We estimated 228,000 (179,000-291,000) HBV-infected adults (≥ 18 years of age) in Germany in 2013, of whom 41% (*n* = 93,000 [52,000–169,000]) were in the general population excluding vulnerable groups. Another 58% (132,000; 126,000-137,000) were migrants, 1.0% (2,400; 900–6,200) PWIO and 0.4% (1,000; 800–1,400) were HIV+MSM. We estimated 214,000 (135,000–340,000) HCV-infected adults in Germany in 2013, of whom 47% (100,000; 38,000–267,000) were in the general population excluding vulnerable groups, 26.0% (56,000; 47,000–66,000) were migrants, 26% (56,000; 50,000–62,000) were PWIO, and 1.0% (2,500; 2,200–2,800) were HIV+MSM, respectively.

**Discussion:**

Our results indicate that more than half of HBV-infected individuals were migrants, and more than half of HCV-infected individuals were PWIO or migrants. This highlights the importance of including relevant subpopulations in national estimates, surveillance, prevention, and therapy. Our estimates serve as a baseline reference for subsequent updates and ongoing monitoring of HBV and HCV epidemiology in Germany.

## 1 Introduction

Viral hepatitis B and C are major global health challenges that contribute to significant morbidity and mortality, mainly due to the chronic course of these infections leading to cirrhosis and hepatocellular carcinoma (1–4). The often-asymptomatic nature of chronic infections allows them to go undetected and progress to advanced stages (5, 6). Worldwide, an estimated 354 million people are living with an HBV or HCV infection (7); 14 million individuals have chronic HBV and 12 million individuals have a chronic HCV infection in the World Health Organization (WHO) European Region alone (7). Despite the availability of effective HBV vaccination since 1986 and direct-acting antivirals that cure over 95% of HCV cases since 2014, viral hepatitis remains a public health problem in the WHO European Region, with 19,000 new HBV and 300,000 new HCV infections annually (8, 9).

With the goal of eliminating HBV and HCV as a public health threat by 2030, WHO emphasizes understanding the epidemic as a critical element of elimination efforts (10). Assessing the national burden of hepatitis, including the prevalence of HBV and HCV infections, is central to guiding public health interventions and decisions within the WHO’s global strategy (10).

Germany is experiencing a concentrated HBV and HCV epidemic, characterized by low prevalence in the general population excluding vulnerable groups, but significantly higher prevalence in several migrant and high-risk populations (11). While HBsAg seroprevalence in the general population ranges from 0.3 to 0.7%, higher prevalence is reported in migrant populations (2.3 to 3.6%), people who inject drugs (PWID) (1.1%) and HIV- positive men who have sex with men (HIV+MSM) (1.2%) (12–16). Similarly, HCV RNA prevalence in the general population ranges from 0.2 to 0.4%, with significantly higher prevalence observed in high-risk populations such as PWID (23 to 54%) and HIV+ MSM (4.2%) (12–16).

To estimate the total number of people living with HBV and HCV in Germany, it is important to consider the prevalence and size of three sectors: the general population excluding vulnerable groups, migrants and high-risk populations. Such estimates were not available. We aimed to fill this gap by providing estimates of the total number of individuals infected with HBV and HCV in Germany and examining their distribution among different population groups in Germany. We also aimed to generate a baseline to monitor the elimination of HBV and HCV in Germany.

## 2 Methods

We estimated the number of people infected with HBV and HCV in Germany in 2013 using a modified workbook method, taking into account the general population excluding vulnerable groups, migrant groups, and specific high-risk populations in Germany: people who inject opioids (PWIOs) and HIV+ MSM. We used serological markers to define people currently infected with HBV and HCV. The migrant populations were also stratified by nationality. Recognizing that many prevalence data sources prior to 2013 were based on anti-HCV, and not on HCV-RNA prevalence, we performed additional calculations using anti-HCV prevalence, to estimate for ever-HCV-infected population and subpopulations, as detailed in the Supplementary Table 1.

Robust prevalence data for the general population excluding vulnerable groups were only available from a study conducted from 2008 to 2011 (17), and the best quality prevalence data in migrant and high-risk populations were available for the time period January 2005 to March 2019 (12, 18). Direct-acting antiviral therapy for HCV became available in Germany in 2014 (19). In the same year, the number of people migrating to Germany substantially increased and the increase continued in the following years (20–22). Therefore, we extrapolated to the situation in Germany in 2013. These estimates serves as a baseline for future estimates.

### 2.1 Definitions for purpose of this study

People infected with HBV: defined as HBsAg-seropositive people, regardless of the stage of the infection (acute or chronic).

People infected with HCV: was defined as HCV-RNA-positive people, regardless of the stage of the infection. If direct measures of viremic infection were missing, it was calculated from anti-HCV seropositivity by applying a factor of 0.74 for spontaneous viral clearance (23). For low and high estimate, we used factor 0.71 and 0.78 accordingly.

People ever-HCV-infected: was defined as anti-HCV seropositive people and comprise people with acute, chronic and cleared HCV infection.

Migrants: were defined as people with non-German citizenship, known nationality and born outside of Germany, who are registered in Germany.

People who inject opioids (PWIO): were defined as a subgroup of PWID and were chosen, because a robust national population size estimate of PWID including non-opioid injectors in Germany was not available.

HIV-positive men who have sex with men (HIV+MSM): was defined as a subgroup of people living with HIV (PLWH) in Germany at increased risk for viral hepatitis (24).

General population excluding vulnerable groups: was defined to include three subpopulations (1) all registered people born in Germany, regardless of nationality; (2) German citizens, regardless of country of birth; (3) people born outside of Germany whose nationality details were unknown. This group excludes the three above mentioned vulnerable population groups: Migrants, PWIO and HIV+MSM.

German citizens born outside of Germany were excluded from the migrant population, as their integration into the German health care system was assumed to be similar to that of people without a migration background. For second-generation migrants (registered people with foreign nationality born in Germany), specific prevalence data were not available, so we excluded them from the migrant population and included them in the general population excluding vulnerable groups.

### 2.2 Workbook method

The workbook method, originally developed to estimate HIV infection in low-endemic countries with concentrated epidemics (25), has been modified to assess HBV and HCV prevalence in risk groups and the general population in the Netherlands (26). We applied this method to calculate the number of people currently infected with HBV and HCV, and to calculating how many people have ever been infected with HCV in Germany. We calculated the total number of people for each subpopulation by multiplying the prevalence point estimates with the population size of the corresponding group.

Estimates include intervals from the lowest to the highest estimated number of infected people in each subpopulation. The lowest estimates were obtained by multiplying the lower limit of the prevalence estimate with the lowest population size estimate for each group. The highest estimates were obtained by multiplying the upper limit of the prevalence estimate with the highest population size estimate. For this process, 95% confidence intervals were used as the lower and upper bounds of the prevalence point estimates, except for PWIO prevalence estimates. For PWIO the mean prevalence and the standard deviation interval were used as lower and upper bounds, based on results of the study (27).

Population size estimates that were based solely on the Federal Statistical Office data were considered accurate and were used without lower and upper boundaries. The workbook was constructed using RStudio and included population size calculations and extrapolations. Tables and graphs were generated in Microsoft Excel (Microsoft Office 2019).

To summarize the estimated prevalences for adult migrant subpopulations and other groups (Table 1) we used the given prevalence estimates to simulate a cross-sectional study stratified into groups. The numbers of participants of this simulated study were constructed to simulate the lower and upper confidence boundaries of the given estimates using the inverse binomial distribution. We tolerated a deviation of 10% for each boundary. We limited the sample size for each group by 10% of the respective population in Germany, and at most at 10,000 individuals, and assumed that the expected proportion of samples was positive in the respective groups. The summarized estimates were then estimated from this simulated cross-sectional study weighted with the inverse inclusion probability (given by the quotient of sample size and population size of the respective groups). An important underlying assumption is that the uncertainties in the different subpopulations are independent of each other.

**Table 1:** Population group specific population size, prevalence and estimated number of HBV and HCV infections.

Population group	HBV		HCV	
Population size (Low-High)	HBsAg prevalence (Low-High)	Number of people with HBV (Low-High)	Viremic HCV prevalence (Low-High)	Number of people with HCV (Low-High)
General population excluding vulnerable groups				
62,200,388	0.15%	93,000	0.16%	100,000
(62,196,150–62,204,326)	(0.08–0.27%)	(52,000–169,000)	(0.06–0.43%)	(38,000–267,000)
Migrants				
5,321,409	2.54%	132,000	1.25	56,000
(5,321,409–5,321,409)	(2.44–2.64%)	(126,000–137,000)	(1.06–1.47)	(47,000–66,000)
PWIO				
126,137	1.88%	2,400	44.00%	56,000
(124,999–127,275)	(0.71–4.90%)	(900–6,200)	(39.35–48.76%)	(50,000–62,000)
HIV+MSM				
44,000	2.33%	1,000	5.67%	2,500
(41,200–47,100)	(1.75–3.10%)	(800–1,400)	(5.01–6.41%)	(2,200–2,800)
Total				
67,691,934	0.34%	228,000	0.32%	214,000
(67,683,758–67,700,120)	(0.27–0.43%)	(179,000–291,000)	(0.20–0.51%)	(135,000–340,000)

HBsAg, Hepatitis B surface antigen; HBV, Hepatitis B virus; HCV, Hepatitis C virus; PWIO, People who inject opioids; HIV+MSM, HIV positive men who have sex with men.

We compared the results from the simulated studies that implied the upper bounds with the ones that implied the lower confidence bounds and checked that they were of the same order of magnitude (Supplementary Table 2). We reported the results of the version that led to more uncertainty in the overall prevalence (Table 1).

### 2.3 Prevalence estimates

For HBV and HCV prevalence data, we used the studies with highest quality identified in the scoping review (11): For HBV, studies reporting HBsAg- seroprevalence in the respective populations were used. For HCV, studies reporting anti-HCV seroprevalence or HCV-RNA in the respective populations were used. Details of the data sources are summarized in Supplementary Table 3.

#### 2.3.1 General population excluding vulnerable groups

To estimate the prevalence of HBV and HCV infections, including all people who have ever been infected with HCV in the adult general population excluding vulnerable groups, we used data from the population-based German Health Interview and Examination Survey for Adults (DEGS1). This survey collected data from adults aged 18 to 79 years between 2008 and 2011 (17) and estimated seroprevalence representative of the adult population in Germany by applying sociodemographic weights. In DEGS1, the seroprevalence among people aged 18 to 79 years was 0.15% (0.08–0.30%) for HBsAg and 0.22% (0.10–0.49%) for anti-HCV and 0.16% (0.06–0.45%) for HCV-RNA ((17); unpublished data). People with non-German citizenship, known nationality and born outside of Germany, who are registered in Germany, were excluded. Other people with a migration background were included in DEGS1, but stratified analysis by nationality and country of birth was not possible due to small case numbers. For calculations in the workbook, DEGS1 participants with a history of migration (migrants according to our definition) were excluded from the DEGS1 estimates, and HBsAg, anti-HCV and HCV RNA prevalence estimates for the population excluding vulnerable groups were recalculated to avoid overlap of subpopulations (Supplementary Table 2).

Since only modeling studies for HBV and HCV prevalence in children were available for the period after 2013 (28–31), we used data from a population-based health survey for children and adolescents, called “KiGGS” conducted from 2003 to 2006 to estimate HBsAg seroprevalence estimates in individuals younger than 18 years (32). Due to the limited power of our HBsAg prevalence calculation and the missing national HBsAg seroprevalence estimates from the respective countries of origin for this age group, we could not stratify the groups by migration status for children and adolescents. Estimates of HCV prevalence in Germany were not available for children and adolescents. Adolescents at increased risk of HCV due to high-risk behaviors were not specifically considered due to lack of data.

#### 2.3.2 Migrant populations

HBV and HCV prevalence data (information about migration or nationality) were not available for specific migrant populations in Germany. Therefore, we used published national prevalence estimates from the respective countries of origin.

For 110 countries, we used national HBsAg seroprevalence estimates from Schweitzer et al. (33), which are reported in detail in Supplementary Table 4. For 7 countries that were not included in Schweitzer et al., we used regional estimates from Kowdley et al. (34).

National estimates of anti-HCV and HCV-RNA prevalence were obtained from Gower et al. (35). For 42 of the 117 countries, only anti-HCV seroprevalence was available, and for a further 42 countries neither national anti-HCV nor HCV-RNA prevalence estimates were available (Supplementary Table 5). For countries without national seroprevalence estimates, we used regional anti-HCV and HCV RNA prevalence estimates from Gower et al. (35). Although regional estimates were not included in our workbook, additional calculations of the number of migrants with HBV and HCV based on regional prevalence estimates are presented separately in Supplementary Tables 6, 7.

#### 2.3.3 People who inject opioids

Prevalence estimates for PWIO were derived from a study on drugs and chronic infectious diseases among people who injected drugs in the last 12 months (27). The study collected data from PWID aged 17–65 years in eight German cities between 2011 and 2014. For prevalence estimates, we used data from a subgroup of this study, specifically those categorized as ‘mainly opioid users in the last 4 weeks’. The mean prevalence estimates for HBsAg, anti-HCV and HCV-RNA from the eight study sites in this group, with the standard deviation, were used in our calculations (Supplementary Table 2 and Table 1).

#### 2.3.4 HIV-positive men who have sex with men

The prevalence of HBsAg, anti-HCV and HCV RNA among HIV+MSM in 2013 was determined using data from a cohort study 1996 to 2019 among individuals with a known date of HIV-seroconversion who were assessed for HBV and HCV-coinfection (Supplementary Table 2 and Table 1). In this dataset, 87% of the study population were MSM (18).

### 2.4 Population size estimates

#### 2.4.1 General population excluding vulnerable groups

The total number of people registered in Germany, and details of age, sex and nationality, was obtained from the Federal Statistical Office (Destatis) on the basis of the 2011 Census. The Central Register of Foreigners (AZR) provides annual data on all non-German nationals in Germany, including information on whether individuals were born abroad (first-generation migrants) or in Germany (second-generation migrants). Both datasets were accessed as GENESIS-Online tables.

For the year 2013, we used census-based data to calculate the size of the general population excluding vulnerable groups. Specifically, we accessed the GENESIS-Online table “12411-0009” (date of record: 31.12.2013; date of query: 16.01.2023) for individuals with German nationality, stratified into the age groups: < 18 years of age and ≥ 18 years of age. We then added the population size of non-German citizens born in Germany (second-generation migrant population) and those born abroad with unknown nationality information for each age group. Finally, we subtracted the population size of estimated PWIOs and HIV+MSM from this population ≥ 18 years of age.

#### 2.4.2 Migrant populations

To determine the number of migrants, we utilized two data sources: census-based data from the Federal Statistical Office accessed through the GENESIS Online tables “12411-0007” and “12411-0009”(date of record: 31.12.2013; date of query: 16.01.2023), and data from the Central Register of Foreigners (AZR) retrieved via the GENESIS Online table “12521-0004” (date of record: 31.12.2013; date of query: 16.01.2023).

The census-based data included all individuals registered in Germany by nationality. Non-German nationals were further categorized into individuals with known nationalities, those with unknown nationalities but identified world regions, stateless persons, and individuals with an unspecified nationality status. For our calculations, we focused on individuals with a known nationality. As the census data did not provide information on migration background, we used data from the AZR to obtain this additional detail. The AZR collects information on individuals with non-German nationality who have been residing in Germany for more than 3 months.

To estimate the number of migrants, we first calculated the proportion of individuals born outside Germany for each nationality among those with known nationality in the AZR. We then applied these proportions to the census-based data for people with known non-German nationality. The resulting population size estimates for each nationality (*n* = 117) in Germany in 2013 can be found in Supplementary Tables 4, 5. The total population size of migrants is presented in Tables 1, 2. Undocumented migrants were not considered for the analyses.

**Table 2:** People in Germany in 2013 by nationality.

Age group (years)	Census-based number of people				AZR-based number of people		extrapolated number of people
	People in Germany	People with German nationality	People with non-German nationality	People with known non-German nationality*	People with known non-German nationality	Migrants with known nationality*	Migrants with known nationality*
< 18	13,075,529	12,242,819	832,710	788,465	821,382	365,420	361,796
≥ 18	67,691,934	61,509,408	6,182,526	5,986,731	6,616,969	5,881,504	5,321,409
Total	80,767,463	73,752,227	7,015,236	6,775,196	7,438,351	6,246,924	5,680,362

AZR, Central Register of Foreign Nationals.

*People with known non-German nationality and exclusion of people with unknown or vague nationality details.

#### 2.4.3 People who inject opioids

For Germany, a robust national estimate was available only for the number of opioid-dependant people in 2016 (36). Furthermore, the PWID population in Europe in 2013 was primarily associated with opiod use (37). For these reasons, we chose the PWIO population as a subgroup of PWID for the calculations. The rate of opioid dependent individuals per 1,000 inhabitants (aged 15 to 64 years) in Germany for 2016 was extrapolated to the population aged 18–64 in the same year. This adjusted rate was applied to the total population aged 18 to 64 years in 2013 (*n* = 50,839,124) to estimate the total number of opioid-dependent people in Germany. To determine the population size of people who inject opioids, we used the proportion of opioid injections (76.9%), as reported in the annual ‘REITOX Report 2013/2014’ by the German Addiction Aid Statistics (38).

#### 2.4.4 HIV-positive men who have sex with men

Estimates of the annual number of people living with HIV (PLWH) and their mode of transmission are published annually, using a back-calculation approach. This involves analyzing annual notification data, reported AIDS/HIV cases, death notifications, death statistics from Destatis (the German Federal Statistical Office) with HIV as the cause of death, and nationwide data on prescribed antiviral drugs (39). For our study, we specifically included HIV+MSM aged 15 to 64 years in 2013, as detailed in Table 1 and Supplementary Table 3.

## 3 Results

### 3.1 Population size estimates

In 2013, Germany had a registered population of 80,767,463, including 67,691,934 adults (≥18 years of age) and 13,075,529 children and adolescents (< 18 years of age). Among adults 6,182,526 had a non-German nationality (Table 2). After excluding people of unknown nationality, we estimated 5,986,731 adults of 117 different nationalities. The AZR recorded on these 6,616,969 adults, confirming that 89% (*n* = 5,881,504) were first-generation immigrants with known nationality, born outside Germany. Applying this proportion to the census-based data, we estimated 5,321,409 adult migrants of known nationality in Germany in 2013 (Table 2 and Table 1).

According to Kraus et al. (36), 3.22 individuals per 1,000 inhabitants aged 18 to 64 were opioid dependent in Germany in 2016. Applying this rate to the population aged 18–64 in 2013, we estimated 164,027 opioid addicts, of whom 77% (*n* = 126,137) were assumed to be PWIO (38) (Table 1).

Based on data extracted from the RKI database of PLWH we extrapolated 44,000 (41,200–47,100) HIV+MSM (Table 1).

After subtracting the number of migrants, PWIO and HIV+MSM from the total adult population (*n* = 67,691,934; Table 2), the general adult population excluding vulnerable groups (≥ 18 years) was 62,200,388 (62,196,150–62,204,326) people (Table 1).

### 3.2 HBV in Germany

**Figure 1:**
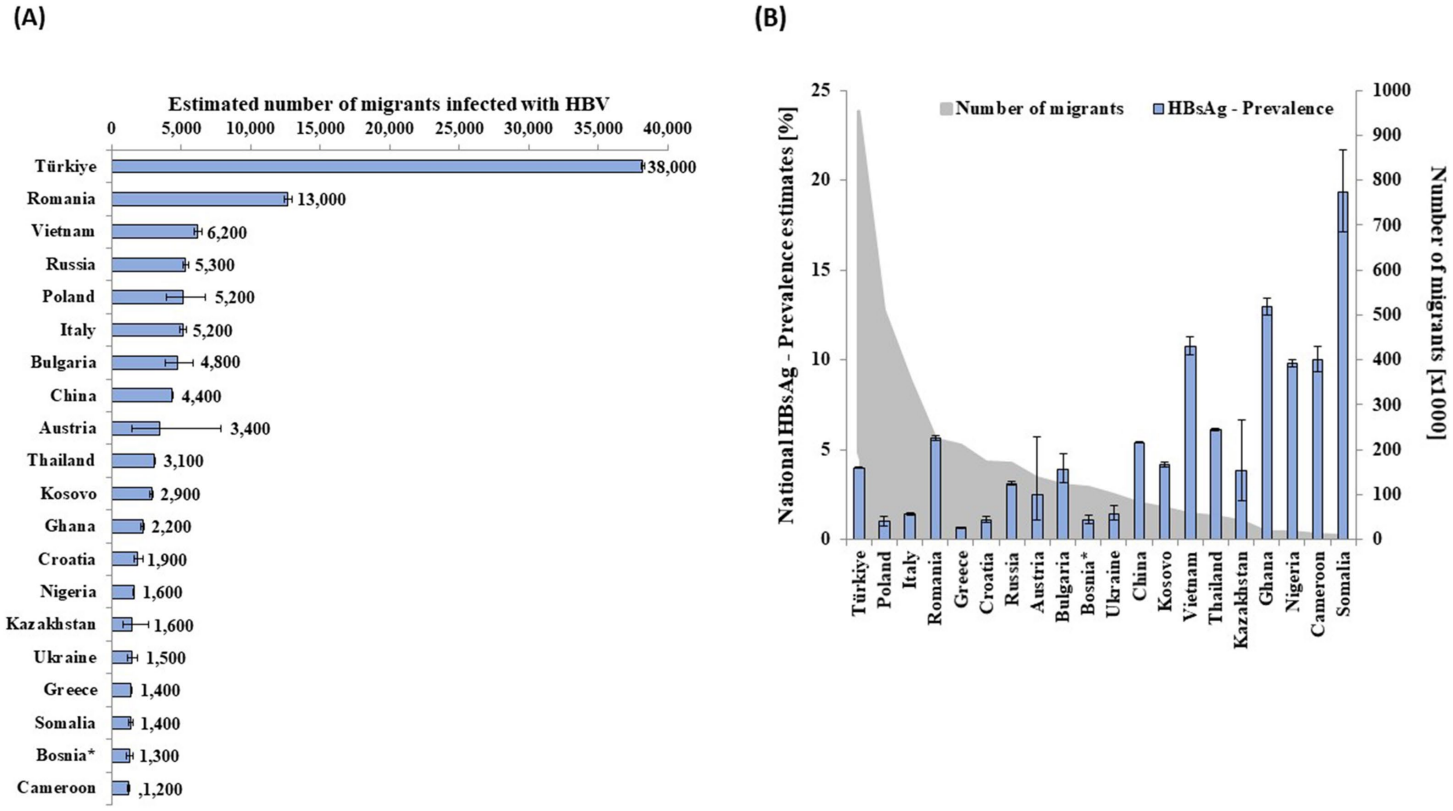
Estimated number of migrants infected with HBV of the largest 20 migrant groups infected with HBV by **(A)** country of nationality and **(B)** respective HBsAg prevalence and population size in Germany in 2013. *Bosnia and Herzegovina.

According to our estimates, 228,000 (179,000–291,000) adults were infected with HBV in Germany in 2013. Table 1 provides detailed data on HBsAg prevalence, population size, and the estimated number of HBV-infected people by subpopulation. With an estimated prevalence of 0.15% (0.08–0.27%) in the general population excluding vulnerable groups, this group accounted for 41% (*n* = 93,000 [52,000–169,000]) of all adults infected with HBV in 2013. The estimated prevalence among children and adolescents (< 18 years of age) was 0.18% (0.11–0.28%), resulting in estimated 24,000 (14,000–37,000) infections across all age groups, without considering migrant status. Migrants accounted for 58% (*n* = 132,000 [126,000–137,000]) of all estimated adults with HBV in Germany (Table 1). PWIO and HIV+MSM with 2,400 (900–6,200) and 1,000 (800–1,400), respectively, accounted for less than 2% of the estimated people with HBV in Germany. Among migrants, the number of people of the 20 most common HBV-infected migrant groups accounted for 80% (*n* = 105,000 of 132,000 migrants) of all HBV cases attributable to migrants (Figure 1). The most frequent nationality was Turkish with estimated 38,200 (38,100–38,400) people, followed by Romanian with 12,700 (12,400–13,000) and Vietnamese with 6,200 (5,900–6,500).

Populations with high prevalence, large population size, or both contributed the most to the total estimate. For example, Polish migrants had a prevalence of 1.01% (0.77–1.32%) and an estimated population size of 511,000 people, resulting in a calculated 5,200 (3,900–6,700) people infected with HBV (Figure 1A and Supplementary Table 4). Vietnamese migrants (*n* = 58,000), with an HBsAg prevalence of 10.76% (10.25–11.28%), were the third most affected migrant population (Figure 1A and Supplementary Table 4). Additional data for 97 nationalities, representing 27,000 migrants with HBV, are shown in Supplementary Table 4.

### 3.3 HCV in Germany

In 2013, we estimated that 214,000 (135,000–340,000) adults in Germany were infected with HCV (Table 1). Of these, 100,000 (38,000–267,000) were part of the general population excluding vulnerable groups, representing 47% of the total HCV-infected population. Both PWIO with an estimated 56,000 (47,000–66,000) HCV-infected people, and migrants with an estimated 56,000 (50,000–62,000) HCV-infected people contributed equally with 26% (Table 1). HIV+MSM with an estimated 2,500 people (2,200–2,800) represented 1% of all estimated HCV-infected people.

**Figure 2:**
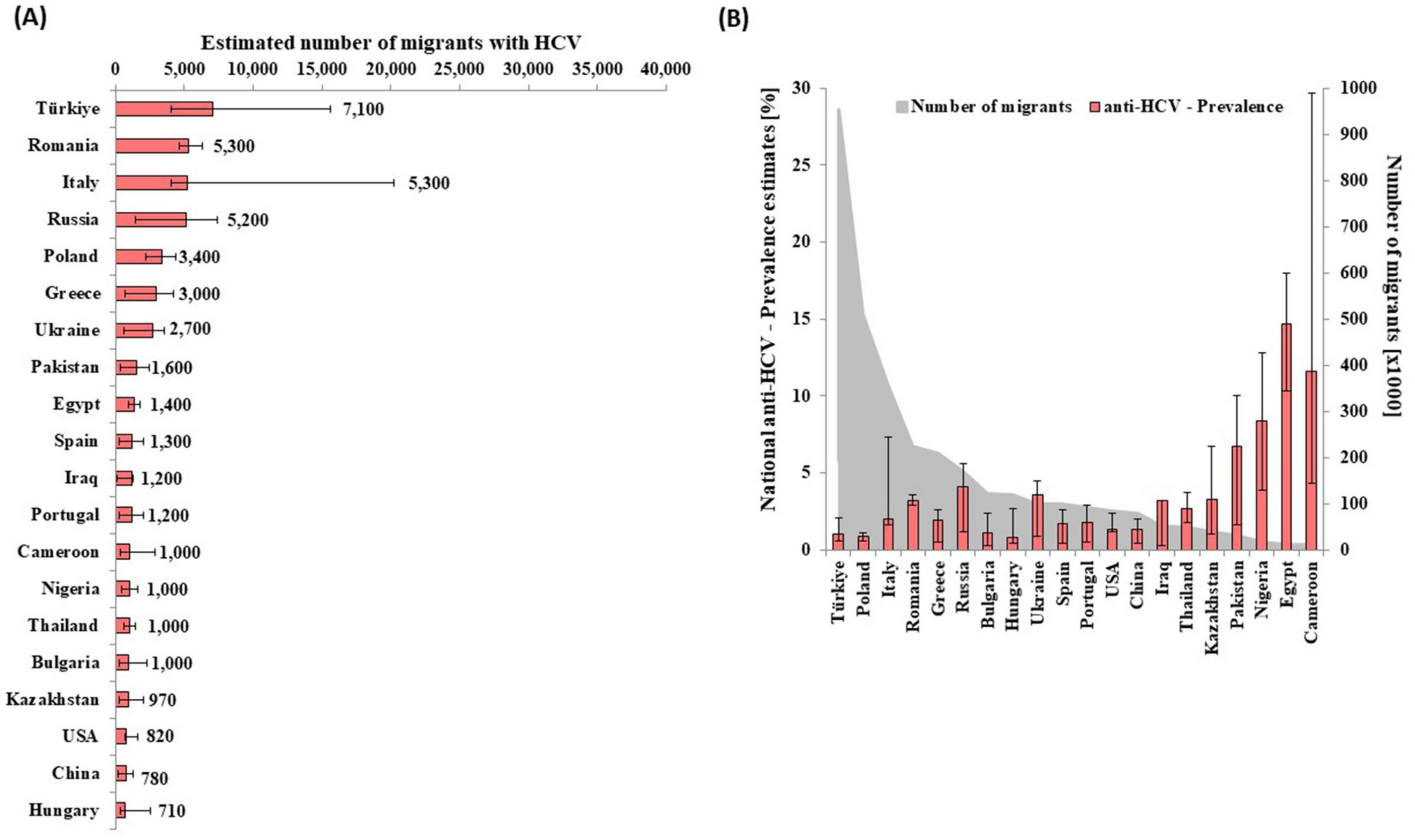
Estimated number of migrants infected with HCV of the largest 20 migrant groups infected with HCV by **(A)** country of nationality and **(B)** respective anti-HCV prevalence and population size **(B)** in Germany in 2013.

Of all adult HCV cases attributable to migrants in 2013, 82% (*n* = 46,000 out of 56,000) were among 20 nationalities, as shown in Figure 2A and. Individuals of Turkish nationality formed the largest group of migrants (*N* = 960,000) and also of HCV-infected migrants (*n* = 7,100 [4,100–16,000]) (Figure 2), followed by Romanians with 5,300 (4,600–6,300) and Italians with 5,300 (4,000–20,200) HCV-infected people. Estimates 97 additional nationalities, corresponding to an estimated 10,000 migrants with HCV, are reported in Supplementary Table 5. Population size, anti-HCV prevalence estimates, and the distribution of ever-infected people among migrant populations in Germany in 2013 are reported and shown in Supplementary Tables 3, 5.

## 4 Discussion

### 4.1 National estimate of people with HBV and HCV in Germany

We applied the workbook method and included the proportions of migrant and high-risk populations, to improve the already published estimates of HBV and HCV prevalence in Germany (11, 12). We were able to demonstrate that 41% of all HBV and 47% of all HCV infections were attributable to the general population excluding vulnerable groups, while 58% of HBV infections were among migrants, and 52% of HCV infections were among PWIO (26%) and migrants (26%) together.

Due to the availability of effective HCV therapy, increasing proportions of people vaccinated and treated for hepatitis B, and changing migration flows over time, our estimates for 2013 should be regarded as a basis to monitor the progress of HBV and HCV elimination in Germany. Our estimates and the resulting prevalence for HBV in 2013 align with the estimates from the Polaris modeling studies in 2016 and 2022 (HBV prevalence: 0.3% [0.2–0.8%] and 0.32% [0.2–0.8%]) for Germany, which are based on data from 2008 to 2011 (29, 30). The older the sources used for prevalence estimates in calculations or modeling, the higher the resulting prevalence. This is evident in the estimates by Schweitzer et al. for Germany, where a HBsAg- prevalence of 1.16% (1.04–1.28%) was estimated for the period 1957–1989, and 0.52% (0.47–0.57%) for 1990–2013 (33). Kowdley et al. estimated the prevalence of chronic HBV among individuals residing in the United States who were born in Germany to be 0.6% (0.4–0.8%), based on HBsAg prevalence data published between 1980 and 2010 (34). The WHO dashboard in its HBV country profiles provides an estimated number of chronic HBV infections of 545,000 (415,000–720,000) for 2015 (28) and these calculations rely on sources from the 1970s and 1980s, which, as seen in the case of Schweitzer et al., yielded in higher prevalence estimates. Apart from the estimates of HBV infected people in Germany in total, our HBV estimate is, to our knowledge, the first national estimate available for Germany that accounts for the prevalence and population size of different populations. Our 2013 estimate for HCV is a bit lower, but still in the range of the 2012 estimate by Bruggmann et al., who modeled the HCV-infected population in Germany at 274,700 (165,000–494,000) infected people (40). More recent estimates used the Multiparameter Evidence Synthesis (MPES) resulting in 196,671 (137,555–279,639) HCV-infected people in 2019 (41). Another modeling study predicted 189,000 [76,700–295,000] HCV-infected people in 2020 (42). Both account for the availability of highly effective direct-acting antivirals and indicate a continuous decline in the number of infected people, consistent with increasing numbers of people tested and treated and rising awareness.

### 4.2 HBV and HCV in the different population groups

#### 4.2.1 General population excluding vulnerable groups

In Germany, the overall HBV and HCV prevalence is low, similar to many other European Union and European Economic Area countries (EU/EFA) (43). After excluding migrants from the whole population according to our definition, we estimated an even lower prevalence, especially for HBV, than reported in DEGS1 (17) (Supplementary Table 2). This can be explained by the substantial share of HBV infections that are attributable to migrants with non-German citizenship, thus representing population not born in Germany, as also shown in our analysis. Due to its large population size with a substantial share of older people (who might have been infected long time ago, the general population excluding vulnerable groups still accounted for 41% of all HBV-infections and 47% of all HCV infections in Germany), emphasizing the need for ongoing prevention, case finding and treatment efforts among this population.

A significant portion of individuals with HBV in the DEGS1 population had German citizenship but were born in Poland, Türkiye, and Kazakhstan. Many with HCV were born in Italy, Romania, and Ukraine. A significant proportion of cases with both HBV and HCV were born in Russia. This highlights the importance of considering history of migration in HBV and HCV surveillance, and in prevention and treatment strategies.

In October 2021, a one-time-screening during regular health check-ups for people 35 years and older was implemented in Germany and may be the reason there is now better case finding (44, 45).

The total number and prevalence of HBV infections in the general population excluding vulnerable groups is expected to continue to decrease due to the universal and targeted hepatitis B vaccination strategy in Germany. We believe that the aging population who acquired HCV many years ago, for example via a blood transfusion before general screening was introduced in 1992 or through former injecting drug use, and who are no longer at risk for infection, is substantially contributing to our estimated number of people with HCV. New serological studies of adults in the population as whole to measure HBV and HCV prevalence are urgently needed to continue to monitor and validate viral hepatitis elimination efforts in Germany.

For HBV in children and adolescents the estimate was based on a study performed in 2003–2006, in which 38.7% of all anti-HBc positive children and adolescents were HBsAg positive (32). Almost three-quarters of all anti-HBc positive children and adolescents had a history of migration (either they were not born in Germany or one or both of their parents were not born in Germany) (32, 46). Same study reports that adolescents aged 14 to 17 years had the highest Anti-HBc prevalence and lowest vaccination rate (32, 47). Only 50% of adolescents with at least one hepatitis B vaccination received the first vaccination before the age of 11 (47). This is in line with the fact that the older age groups in the population-based health survey for children and adolescents were born before the introduction of universal hepatitis B vaccination in 1995 and before introducing screening of pregnant women for HBsAg in 1994 (32). Therefore, our calculated number of children affected by hepatitis B is likely an overestimation. In a more recent study of under-18-years-olds conducted between 2014 and 2017 of more than 3,000 children, there were zero cases found with chronic or acute hepatitis B infection (personal communication, Manuscript in preparation). This indicates that Germany has made significant efforts in hepatitis B control and has even reached elimination of mother-to-child transmission impact indicators (personal communication). Given the high risk of chronic HBV development in children (5), early prevention interventions, including prevention of mother-to-child transmission, especially for pregnant women from high-prevalence countries, should be monitored and improved if needed.

#### 4.2.2 Migrant populations

In 2013, migrants made up a substantial proportion of the HBV and HCV infections in Germany. The Turkish and Romanian migrant subpopulations were the largest contributors, with high prevalences of both HBV and HCV and substantial subpopulation sizes. Other notable subpopulations included Russians and Italians, each with similar numbers for HBV and HCV. Subpopulations from African and Asian countries, despite their small size, also made a significant contribution to the overall prevalence, due to high prevalence estimates.

The results for 2013 are consistent with ECDC reports and reflect the European situation at that time (48). We could not include registered people with unknown or unclear nationality details into our calculations (*n* = 196,000; Table 2) as well as estimated 200.000–500.000 undocumented migrants as proposed in several sources (49, 50). Overlap with other at-risk populations like PWIO and sex worker is possible.

As migration, along with both registered and undocumented migrants, has increased significantly in Europe and Germany since 2013 (51–53) our estimates provide a baseline for needed updated calculations to account for example for significant refugee migration to Germany in 2014/15 from Syria and 2022/23 from Ukraine (45, 54).

The high infection rates among migrants in 2013, combined with shifts in migration patterns, underscore the need for tailored HBV and HCV prevention, testing, and treatment options that are easily accessible even for people who have recently arrived to Germany, have language barriers, or lack legal status and health insurance, and who therefore have limited access to health care services (55, 56).

All pregnant women, irrespective of health insurance and legal status, should receive timely antenatal care. For HBV positive pregnant women, measures for prevention of mother-to child transmission of hepatitis B should be undertaken. This includes providing HBV therapy for pregnant women with a high HBV viral load, as well as timely passive and active immunization for the newborn.

#### 4.2.3 People who inject opioids

PWIO in Germany accounted for 26% of all HCV and 1% of HBV infections in 2013. Despite representing only 0.19% of the German population in 2013, PWID have the highest HCV prevalence, in line with European trends (43, 57, 58). Globally, around 40% of HCV infections are attributed to injecting drug use, and that may be as high as 80% for Western Europe (57, 58). Our estimates are in line with previous German data (59) and consistent with French findings (60).

While our results may underestimate the impact of drug use due to the focus on people who inject opioids, the role of injecting drug use in HCV epidemiology is likely to be more significant. According to the recent MPES study by Thomadakis et al., 49% of viremic HCV-infections in Germany were attributable to injecting drug use (41), however this estimate included both, current and previous injecting drug use, whereas our estimate is only in current injectors (and only of opioids).

Nonetheless, our estimates demonstrate the importance of scaling up HCV testing and referral to treatment for PWID as both free of charge and easily accessible to all, complemented by prevention measures such as access to clean consumption materials and opioid substitution (61). Since the year of our estimate, the epidemiological situation has changed, due to the availability of effective treatment, in particular for HCV, and increased screening, as seen in a recent German pilot study (63), in France (60) and reported in recent modeling studies (42, 62).

While the prevalence of chronic HBV infection in the PWIO population remains moderate, substantially higher Anti-HBc prevalence, which indicates a past infection, suggests a continuous need for prevention through vaccination (27, 63). Despite existing indications for vaccination and recommendations by the German standing vaccination committee (STIKO) (64), fewer than 50% of people in this group have HBs antibodies in sufficient titre detectable as a proof of effective vaccination (63). Therefore, prevalence estimates to monitor the impact of HCV treatment and HBV vaccination in this vulnerable group are essential to monitor trends. Results from a pilot study of PWID indicate a decrease in viremic HCV prevalence among study participants in two federal states, but need to be verified in a more robust study (63). For better HCV and HBV prevalence calculations in the future to improve strategic planning, a PWID population size estimate is urgently needed.

#### 4.2.4 HIV-positive men who have sex with men

HIV+MSM accounted for a small proportion of the HBV- and HCV-infected population in our study, with an estimated 0.4% of all HBV-infected and 1% of all HCV-infected people in 2013, due to their relatively small population size estimate. Since then, the epidemiological situation might have changed, with changes in risk behaviors, increasing awareness of viral hepatitis, or increased case-finding. MSM with sexual and drug-related risk behaviors are at particularly high risk for HCV, and should be tested for viral hepatitis at least annually (65, 66), treated if diagnosed, and should be vaccinated against HBV (64). Despite existing recommendations, the self-reported vaccination coverage remains at less than 50% among MSM (67). As more MSM are diagnosed with HIV (68) and there is greater support for HIV-positive people in treatment, it is important to examine whether efforts such as free HBV vaccination services and related campaigns for MSM have reduced the number of HIV+MSM with HBV or HCV since 2013 (67, 69). Updated estimates, based on our calculations, can help to clarify the impact of these interventions.

### 4.3 Limitations

Despite using the best quality data available, various biases may affect our estimates. The workbook approach, although carefully applied, may still include overlapping subpopulations, introducing potential bias. As there is a low prevalence and limited sample size in DEGS1, and the measured prevalence has wide confidence intervals, our estimates of the infected population resulted in a wide confidence interval around the point estimate.

Applying DEGS1 prevalences to age groups over 79 years that were not covered by DEGS1 may have overestimated the number of infected people in that age group. Access to robust prevalence data would reduce this limitation. Data for children were derived from a study conducted between 2003 and 2006; this may have led to overestimating the number of children and adolescents with hepatitis B in Germany in 2013. A further limitation was that we had to rely on country prevalence estimates for countries of migrant nationality. To limit the impact of this bias, we selected high quality prevalence estimates for countries where available, and only used modeling data in countries where no better estimate was published. This might have led to under- or over-estimating the number of HBV and HCV infections among migrant populations. Therefore, the calculations should be updated as new, higher quality evidence arises.

In the absence of a subpopulation estimate for PWID, we used the subpopulation of PWIO, resulting in a gap in the prevalence estimates for drug users.

Due to missing population size or prevalence data high-risk populations including blood transfusion recipients before 1991, people living with HIV (HIV+MSM excluded), sex workers, people in prisons and other closed settings as well as asylum seekers, undocumented migrants were not included in our analysis. Furthermore, our definition of the migrant population used for the purposes of this study does not entirely correctly reflect populations with a history of migration in Germany.

Finally, for HCV, prevalence estimates for viremic infection were missing for some countries of origin for the migrant population. We therefore had to rely on antibody prevalence and apply a factor to come up with an estimate for viremic infection prevalence for the respective country of origin. This resulted in further uncertainty in the HCV estimate.

## 5 Conclusion

This is the first study in Germany that has estimated the total number of people infected with HBV and HCV by considering the virus prevalences in each subpopulation. Our estimates highlight the role of specific migrant and at-risk populations in the epidemiology of HBV and HCV in Germany. This confirms the importance of targeted interventions for various subpopulations, including vaccination, testing, and treatment. At the same time, the general population excluding vulnerable groups remains an important target group despite very low prevalence.

Our historical estimates serve as a baseline for ongoing monitoring of elimination efforts in Germany. While the workbook method has proved feasible, its accuracy depends on the availability and comparability of data. When repeating similar estimations in the future, we recommend including up-to-date prevalence estimates, more accurate estimates of population size for PWID, people in prisons, and other subpopulations not covered in the current analysis.

## Data Availability

The original contributions presented in the study are included in the article/Supplementary material, further inquiries can be directed to the corresponding author.

## References

[1] Duarte G Williams CJ Vasconcelos P Nogueira P. Capacity to report on mortality attributable to chronic hepatitis B and C infections by member states: An exercise to monitor progress towards viral hepatitis elimination. J Viral Hepat. (2018) 25:878–82. doi: 10.1111/jvh.12882, PMID: 29479771

[2] WHO. (2024). Hepatitis B. Fact sheets. Available online at: https://www.who.int/news-room/fact-sheets/detail/hepatitis-b (Accessed May 24, 2024).

[3] WHO. (2024). Hepatitis C. Fact sheets. Available online at: https://www.who.int/news-room/fact-sheets/detail/hepatitis-b (Accessed May 24, 2024).

[4] World Health Organization. Global hepatitis report 2017. Geneva: World Health Organization (2017).

[5] Ganem D Prince AM. Hepatitis B virus infection--natural history and clinical consequences. N Engl J Med. (2004) 350:1118–29. doi: 10.1056/NEJMra031087, PMID: 15014185

[6] Thein HH Yi Q Dore GJ Krahn MD. Estimation of stage-specific fibrosis progression rates in chronic hepatitis C virus infection: a meta-analysis and meta-regression. Hepatology. (2008) 48:418–31. doi: 10.1002/hep.22375, PMID: 18563841

[7] World Health Organization. (2023). Hepatitis. Health topics. Available online at: https://www.who.int/health-topics/hepatitis#tab=tab_1 (Accessed May 24, 2024).

[8] World Health Organization. (2022). Hepatitis B in the WHO European region. Fact sheet 2022. Available online at: https://www.who.int/europe/publications/m/item/hepatitis-b-in-the-who-european-region-factsheet-july-2022 (Accessed May 24, 2024).

[9] World Health Organization. (2022). Hepatitis C in the WHO European region. Fact sheet 2022. Available online at: https://www.who.int/europe/publications/hepatitis-c-in-the-who-european-region-factsheet-july-2022 (Accessed May 24, 2024).

[10] World Health Organization. Global health sector strategy on viral hepatitis 2016–2021. Geneva: World Health Organization (2016).

[11] Steffen G Sperle I Leendertz SA Sarma N Beermann S Thamm R. The epidemiology of hepatitis B, C and D in Germany: a scoping review. PLoS One. (2020) 15:e0229166. doi: 10.1371/journal.pone.0229166, PMID: 32150561 PMC7062254

[12] Sperle I Steffen G Leendertz SA Sarma N Beermann S Thamm R. Prevalence of hepatitis B, C, and D in Germany: results from a scoping review. Front Public Health. (2020) 8:424. doi: 10.3389/fpubh.2020.00424, PMID: 33014960 PMC7493659

[13] Heidrich B Cetindere A Beyaz M Stahmeyer JT Basaran MM Braynis B. High prevalence of hepatitis markers in immigrant populations: a prospective screening approach in a real-world setting. Eur J Gastroenterol Hepatol. (2014) 26:1090–7. doi: 10.1097/MEG.0000000000000164, PMID: 25076065

[14] Wolffram I Petroff D Bätz O Jedrysiak K Kramer J Tenckhoff H. Prevalence of elevated ALT values, HBsAg, and anti-HCV in the primary care setting and evaluation of guideline defined hepatitis risk scenarios. J Hepatol. (2015) 62:1256–64. doi: 10.1016/j.jhep.2015.01.011, PMID: 25617500

[15] Bert F Rindermann A Abdelfattah MA Stahmeyer JT Rossol S. High prevalence of chronic hepatitis B and C virus infection in a population of a German metropolitan area: a prospective survey including 10 215 patients of an interdisciplinary emergency unit. Eur J Gastroenterol Hepatol. (2016) 28:1246–52. doi: 10.1097/MEG.0000000000000702, PMID: 27439034

[16] Hampel A Solbach P Cornberg M Schmidt RE Behrens GM Jablonka A. Current seroprevalence, vaccination and predictive value of liver enzymes for hepatitis B among refugees in Germany. Bundesgesundheitsblatt Gesundheitsforschung Gesundheitsschutz. (2016) 59:578–83. doi: 10.1007/s00103-016-2333-8, PMID: 27090247

[17] Poethko-Muller C Zimmermann R Hamouda O Faber M Stark K Ross RS. Epidemiology of hepatitis a, B, and C among adults in Germany: results of the German health interview and examination survey for adults (DEGS1). Bundesgesundheitsblatt Gesundheitsforschung Gesundheitsschutz. (2013) 56:707–15. doi: 10.1007/s00103-013-1673-x, PMID: 23703489

[18] Krings A Schmidt D Meixenberger K Bannert N Munstermann D Tiemann C. Decreasing prevalence and stagnating incidence of hepatitis C-co-infection among a cohort of HIV-1-positive patients, with a majority of men who have sex with men, in Germany, 1996-2019. J Viral Hepat. (2022) 29:465–73. doi: 10.1111/jvh.13670, PMID: 35302675

[19] Sarrazin C Zimmermann T Berg T Neumann UP Schirmacher P Schmidt H. Prophylaxis, diagnosis and therapy of hepatitis-C-virus (HCV) infection: the German guidelines on the management of HCV infection - AWMF-register-no.: 021/012. Z Gastroenterol. (2018) 56:756–838. doi: 10.1055/a-0599-1320, PMID: 29945279

[20] Flüchtlinge BfMu. Migrationsbericht 2021. Germany: Migrationsbericht (2023).

[21] Flüchtlinge BfMu. Migrationsbericht 2014. Germany: Migrationsbericht (2016).

[22] Flüchtlinge BfMu. Migrationsbericht 2015. Germany: Migrationsbericht (2016).

[23] Micallef JM Kaldor JM Dore GJ. Spontaneous viral clearance following acute hepatitis C infection: a systematic review of longitudinal studies. J Viral Hepat. (2006) 13:34–41. doi: 10.1111/j.1365-2893.2005.00651.x, PMID: 16364080

[24] Vaux S Chevaliez S Saboni L Sauvage C Sommen C Barin F. Prevalence of hepatitis C infection, screening and associated factors among men who have sex with men attending gay venues: a cross-sectional survey (PREVAGAY), France, 2015. BMC Infect Dis. (2019) 19:315. doi: 10.1186/s12879-019-3945-z, PMID: 30971207 PMC6458747

[25] Walker N Stover J Stanecki K Zaniewski AE Grassly NC Garcia-Calleja JM. The workbook approach to making estimates and projecting future scenarios of HIV/AIDS in countries with low level and concentrated epidemics. Sex Transm Infect. (2004) 80:i10–3. doi: 10.1136/sti.2004.010207, PMID: 15249693 PMC1765837

[26] Koopsen J van Steenbergen JE Richardus JH Prins M Op de Coul ELM Croes EA. Chronic hepatitis B and C infections in the Netherlands: estimated prevalence in risk groups and the general population. Epidemiol Infect. (2019) 147:e147. doi: 10.1017/S0950268819000359, PMID: 30869044 PMC6518512

[27] Robert Koch Institute. Abschlussbericht der Studie “Drogen und chronischen Infektionskrankheiten in Deutschland” (DRUCK-Studie). Berlin: Robert Koch Institute (2016).

[28] WHO. (2024). Dashboard: HBV country profiles. Available online at: https://situatedlaboratories.net/who-hepB-dashboard/src/.

[29] Polaris Observatory Collaborators. Global prevalence, treatment, and prevention of hepatitis B virus infection in 2016: a modelling study. Lancet Gastroenterol Hepatol. (2018) 3:383–403. doi: 10.1016/S2468-1253(18)30056-6, PMID: 29599078

[30] Polaris Observatory Collaborators. Global prevalence, cascade of care, and prophylaxis coverage of hepatitis B in 2022: a modelling study. Lancet Gastroenterol Hepatol. (2023) 8:879–907. doi: 10.1016/S2468-1253(23)00197-8, PMID: 37517414

[31] Schmelzer J Dugan E Blach S Coleman S Cai Z DePaola M. Global prevalence of hepatitis C virus in children in 2018: a modelling study. Lancet Gastroenterol Hepatol. (2020) 5:374–92. doi: 10.1016/S2468-1253(19)30385-1, PMID: 31954439

[32] Cai W Poethko-Muller C Hamouda O Radun D. Hepatitis B virus infections among children and adolescents in Germany: migration background as a risk factor in a low seroprevalence population. Pediatr Infect Dis J. (2011) 30:19–24. doi: 10.1097/INF.0b013e3181ef22d5 20683220

[33] Schweitzer A Horn J Mikolajczyk RT Krause G Ott JJ. Estimations of worldwide prevalence of chronic hepatitis B virus infection: a systematic review of data published between 1965 and 2013. Lancet. (2015) 386:1546–55. doi: 10.1016/S0140-6736(15)61412-X 26231459

[34] Kowdley KV Wang CC Welch S Roberts H Brosgart CL. Prevalence of chronic hepatitis B among foreign-born persons living in the United States by country of origin. Hepatology. (2012) 56:422–33. doi: 10.1002/hep.24804, PMID: 22105832

[35] Gower E Estes C Blach S Razavi-Shearer K Razavi H. Global epidemiology and genotype distribution of the hepatitis C virus infection. J Hepatol. (2014) 61:S45–57. doi: 10.1016/j.jhep.2014.07.027, PMID: 25086286

[36] Kraus L Seitz NN Schulte B Cremer-Schaeffer P Braun B Verthein U. Estimation of the number of people with opioid addiction in Germany. Dtsch Arztebl Int. (2019) 116:137–43. doi: 10.3238/arztebl.2019.0137, PMID: 30961791 PMC6460011

[37] EMCDDA. European drug report 2014: Trends and developments. Luxembourg: Publications Office of the European Union (2014).

[38] Pfeiffer-Gerschel TJL Stumpf D Budde A Rummel C Casati A. Bericht 2014 des nationalen REITOX-Knotenpunkts an die EBDD. München: Deutsche Beobachtungsstelle für Drogen und Drogensucht DBDD (2014).

[39] der Heiden AM Marcus U Kollan C Schmidt D. Schätzung der Zahl der HIV-Neuinfektionen und der Gesamtzahl von Menschen mit HIV in Deutschland. Stand Ende 2017. Epid Bull. (2018) 47:509–22. doi: 10.17886/EpiBull-2018-056

[40] Bruggmann P Berg T Ovrehus AL Moreno C Brandao Mello CE Roudot-Thoraval F. Historical epidemiology of hepatitis C virus (HCV) in selected countries. J Viral Hepat. (2014) 21:5–33. doi: 10.1111/jvh.12247, PMID: 24713004

[41] Thomadakis C Gountas I Duffell E Gountas K Bluemel B Seyler T. Prevalence of chronic HCV infection in EU/EEA countries in 2019 using multiparameter evidence synthesis. Lancet Reg Health Eur. (2024) 36:100792. doi: 10.1016/j.lanepe.2023.100792, PMID: 38188273 PMC10769889

[42] Tergast TL Blach S Tacke F Berg T Cornberg M Kautz A. Updated epidemiology of hepatitis C virus infections and implications for hepatitis C virus elimination in Germany. J Viral Hepat. (2022) 29:536–42. doi: 10.1111/jvh.13680, PMID: 35357770

[43] ECDC. Hepatitis B and C epidemiology in selected population groups in the EU/EEA. Stockholm: ECDC (2018).

[44] Biallas R Zimmermann R Dudareva S. Comment on "population-based screening works: effect of integrating screening for hepatitis B and C into the general health check-up in Germany". J Hepatol. (2024) 81:e28–9. doi: 10.1016/j.jhep.2024.02.013, PMID: 38403028

[45] Biallas RSG Burdi S Diercke M Dörre A Méndez-Brito A Sievers C. Anstieg der übermittelten Hepatitis-B-und Hepatitis-C-Fälle in Deutschland im Jahr 2022. Epid Bull. (2023) 31:3–16. doi: 10.25646/11669

[46] Schenk L Ellert U Neuhauser H. Children and adolescents in Germany with a migration background. Methodical aspects in the German Health Interview and Examination Survey for Children and Adolescents (KiGGS). Bundesgesundheitsblatt Gesundheitsforschung Gesundheitsschutz. (2007) 50:590–9. doi: 10.1007/s00103-007-0220-z 17514443

[47] Poethko-Müller C Kuhnert R Schlaud M. Durchimpfung und Determinanten des Impfstatus in Deutschland-Ergebnisse des Kinder- und Jugendgesundheitssurveys (KiGGS). Bundesgesundheitsblatt Gesundheitsforschung Gesundheitsschutz. (2007) 50:851–62. doi: 10.1007/s00103-007-0248-0, PMID: 17514471

[48] ECDC. Epidemiological assessment of hepatitis B and C among migrants in the EU/EEA. Stockholm: ECDC (2016).

[49] Kovacheva V. Irregular migration in Germany since the turn of the millennium-development, economic background and discourses. Hamburg: Institute of International Economics (HWWI), Database on Irregular Migration (2010).

[50] Vogel D. (2015).Update report Germany: Estimated number of irregular foreign residents in Germany (2014). Database on irregular migration, update report. Available online at: http://irregular-migrationnet (Accessed December 5, 2024).

[51] IOM. (2024). World migration report 2024. Available online at: https://publications.iom.int/books/world-migration-report-2024 (Accessed December 5, 2024).

[52] Flüchtlinge BfMu. Migrationsbericht 2022. Germany: Migrationsbericht (2024).

[53] Connor PPJS. Europe’s unauthorized immigrant population peaks in 2016, Then Levels Off. Washington, DC: Pew Research Cente (2019).

[54] von Laer A Diercke M An der Heiden M Altmann D Zimmermann R Dudareva S. Implications of a change in case definition and screening of asylum seekers for hepatitis B surveillance in Germany in 2015 and 2016. Epidemiol Infect. (2020) 148:e36. doi: 10.1017/S0950268820000242, PMID: 32089143 PMC7058648

[55] EASL WHO, ECDC. Ensuring high-quality viral hepatitis care for refugees from Ukraine. Geneva: EASL WHO, ECDC (2022).

[56] Kim JU Ingiliz P Shimakawa Y Lemoine M. Improving care of migrants is key for viral hepatitis elimination in Europe. Bull World Health Organ. (2021) 99:280–6. doi: 10.2471/BLT.20.260919, PMID: 33953445 PMC8085634

[57] Grebely J Larney S Peacock A Colledge S Leung J Hickman M. Global, regional, and country-level estimates of hepatitis C infection among people who have recently injected drugs. Addiction. (2019) 114:150–66. doi: 10.1111/add.14393, PMID: 30035835 PMC6657799

[58] Trickey A Fraser H Lim AG Peacock A Colledge S Walker JG. The contribution of injection drug use to hepatitis C virus transmission globally, regionally, and at country level: a modelling study. Lancet Gastroenterol Hepatol. (2019) 4:435–44. doi: 10.1016/S2468-1253(19)30085-8, PMID: 30981685 PMC6698583

[59] Razavi H Waked I Sarrazin C Myers RP Idilman R Calinas F. The present and future disease burden of hepatitis C virus (HCV) infection with today's treatment paradigm. J Viral Hepat. (2014) 21:34–59. doi: 10.1111/jvh.12248, PMID: 24713005

[60] Brouard C Pillonel J Boussac M de Ledinghen V Rachas A Silvain C. French hepatitis C care cascade: substantial impact of direct-acting antivirals, but the road to elimination is still long. BMC Infect Dis. (2020) 20:759. doi: 10.1186/s12879-020-05478-6, PMID: 33059617 PMC7559725

[61] Blach S Schaetti C Bruggmann P Negro F Razavi H. Cost-effectiveness analysis of strategies to manage the disease burden of hepatitis C virus in Switzerland. Swiss Med Wkly. (2019) 149:w20026. doi: 10.4414/smw.2019.20026 30905063

[62] Polaris Observatory HCVC. Global change in hepatitis C virus prevalence and cascade of care between 2015 and 2020: a modelling study. Lancet Gastroenterol Hepatol. (2022) 7:396–415. doi: 10.1016/S2468-1253(21)00472-6, PMID: 35180382

[63] Steffen G Krings A Guttmann S Lübke N Meyer-Schlinkmann K Tiemann C. DRUCK 2.0-study group. Progress and challenges in the elimination of hepatitis C among people who inject drugs in Germany: results of a pilot study for a national monitoring system, 10 years after the first data collection. Harm Reduct J. (2024) 21:222. doi: 10.1186/s12954-024-01119-2 39707505 PMC11660851

[64] Robert Koch-Institute. Ständige Impfkommission: Empfehlungen der Ständigen Impfkommission (STIKO) beim Robert Koch-Institute 2024. Epid Bull. (2024) 2024:1–72. doi: 10.25646/11892.4

[65] ECFDPA. Public health guidance on HIV, hepatitis B and C testing in the EU/EEA. Stockholm: ECFDPA (2018).

[66] Recently acquired and early chronic hepatitis C in MSM: recommendations from the European treatment network for HIV, hepatitis and global infectious diseases consensus panel. AIDS. (2020) 34:1699–711. doi: 10.1097/QAD.0000000000002622, PMID: 32694411

[67] Brandl M Schmidt A Marcus U An der Heiden M Dudareva S. Are men who have sex with men in Europe protected from hepatitis B? Epidemiol Infect. (2020) 148:163. doi: 10.1017/S0950268820000163, PMID: 32052715 PMC7026898

[68] Robert Koch-Institute. Schätzung der Zahl der HIV-Neuinfektionen und der Gesamtzahl von Menschen, die mit HIV in Deutschland leben. Epid Bull. (2019) 48:3–16. doi: 10.25646/7213

[69] Brandl M Schmidt AJ Marcus U Duffell E Severi E Mozalevskis A. Self-reported hepatitis a and B vaccination coverage among men WHO have sex with men (MSM), associated factors and vaccination recommendations in 43 countries of the WHO European region: results from the European MSM internet survey, EMIS-2017. Euro Surveill. (2024) 29:100. doi: 10.2807/1560-7917.ES.2024.29.45.2400100, PMID: 39512170 PMC11544724

