# Supplementary material for: Estimated number of people infected with hepatitis B and C virus in Germany in 2013: a baseline prevalence estimate using the workbook method
Source: Front Public Health. 2025 Apr 7;13:1471256. doi: 10.3389/fpubh.2025.1471256 (PMC12009770; doi:10.3389/fpubh.2025.1471256)
Supplement: Supplementary file 1 [file Table_1.docx]

**Supplementary Table 1:** Subpopulation specific population size, prevalence and number of ever-HCV-infected people

| **Subpopulations** | | **Ever-HCV-infected** | |
| --- | --- | --- | --- |
| **Age range**  **(years)** | **Population size**  **(Low–High)** | **anti-HCV prevalence**  **(Low–High)** | **Number of ever-HCV infected people**  **(Low–High)** |
| **General population excluding vulnerable groups** | | | |
| ≥ 18 | 62,200,388 | 0.22% | 135,000 |
|  | (62,196,150–62,204,326) | (0.10–0.45%) | (64,000–282,000) |
| **Migrants** | | | |
| ≥ 18 | 5,321,409 | 1.69% | 75,000 |
|  | (5,321,409–5,321,409) | (1.43–1.99%) | (64,000–88,000) |
| **PWIO** | | | |
| 18-64 | 126,137 | 64% | 81,000 |
|  | (124,999–127,275) | (60.66–67.21%) | (77,000–85,000) |
| **HIV+MSM** | | | |
| 18-64 | 44,000 | 9.78% | 4,300 |
|  | (41,200–47,100) | (8.94%–10.69%) | (3,900–4,700) |
| **Total** | | | |
| **≥ 18** | **67,691,934** | **0.44%** | **295,000** |
|  | **(67,683,758–67,700,120)** | **(0.31**–**0.62%)** | **(209,000**–**415,000)** |

HBsAg, Hepatitis B surface antigen; HBV, Hepatitis B virus; HCV, Hepatitis C virus; PWIO, People who inject opioids; HIV+MSM, HIV positive men who have sex with men.
