# Supplementary material for: Estimated number of people infected with hepatitis B and C virus in Germany in 2013: a baseline prevalence estimate using the workbook method
Source: Front Public Health. 2025 Apr 7;13:1471256. doi: 10.3389/fpubh.2025.1471256 (PMC12009770; doi:10.3389/fpubh.2025.1471256)
Supplement: Supplementary file 2 [file Table_2.docx]

**Supplementary Table 2:** Viral prevalence estimates based on a cross–sectional simulation

| **HBsAg prevalence estimates (Low–High)** | | | **anti–HCV prevalence estimates (Low–High)** | | | **Viremic HCV prevalence estimates**  **(Low–High)** | | |
| --- | --- | --- | --- | --- | --- | --- | --- | --- |
| **Data Source (from literature)** | **SCS – lower confidence bound** | **From IBD with upper confidence bound** | **From Data Source** | **From IBD with lower confidence bound** | **From IBD with upper confidence bound** | **From Data Source** | **From IBD with lower confidence bound** | **From IBD with upper confidence bound** |
| **General population excluding vulnerable groups (****≥ 18 years)** | | |  |  |  |  |  |  |
| 0.15  (0.08–0.30) | 0.15  (0.09–0.25) | 0.15  (0.08–0.29) | 0.22  (0.10–0.49) | 0.22  (0.12–0.40) | 0.22  (0.10–0.45) | 0.16*  (0.06–0.45) | 0.16*  (0.08–0.34) | 0.16*  (0.06–0.43) |
| **Migrants (≥ 18 years)** | | |  |  |  |  |  |  |
| – | 2.54  (0.44–2.63) | 2.54  (0.44–2.64) | – | 1.69  (1.46–1.95) | 1.69  (1.43–1.99) | – | 1.25  (1.08–1.45) | 1.25  (1.06–1.47) |
| **PWIO (≥ 18 years)** | | |  |  |  |  |  |  |
| 1.88  (0.70–4.90) | 1.88  (0.90–3.89) | 1.88  (0.71–4.90) | 64.00  (47.50–74.00) | 63.64  (53.13–72.99) | 64.00  (60.66–67.21) | 43.95*  (24.60–53.70) | 44.12*  (28.63–60.84) | 44.00*  (39.35–48.76) |
| **HIV+MSM (≥ 18 years)** | | |  |  |  |  |  |  |
| 2.3  (0.15–3.41) | 2.33  (0.33–14.75) | 2.33  (1.75–3.10) | 9.78  (8.29–11.49) | 9.87  (8.94–10.69) | 9.87  (8.94–10.69) | 5.67*  (4.55–7.05) | 5.67*  (5.02–6.39) | 5.67*  (5.01–6.41) |
| **Total (≥ 18 years)** | | |  |  |  |  |  |  |
| – | 0.34  (0.27–0.42) | 0.34  (0.2–0.44) | – | 0.44  (0.33–0.59) | 0.44  (0.31–0.62) |  | 0.32  (0.22–0.46) | 0.32  (0.20–0.51) |

HBsAg, Hepatitis B surface antigen; HBV, Hepatitis B virus; HCV, Hepatitis C virus; PWIO, People who inject opioids; HIV+MSM, HIV positive men who have sex with men; SCS, simulated cross–sectional study; IBD, inverse binomial distribution

* based on HCV–RNA prevalence estimates

Caption: Prevalences estimated using a simulated cross–sectional study, directly from published data (not available for combined population groups e.g. migrants)
