# Supplementary material for: Estimated number of people infected with hepatitis B and C virus in Germany in 2013: a baseline prevalence estimate using the workbook method
Source: Front Public Health. 2025 Apr 7;13:1471256. doi: 10.3389/fpubh.2025.1471256 (PMC12009770; doi:10.3389/fpubh.2025.1471256)
Supplement: Supplementary file 3 [file Table_3.docx]

**Supplementary Table 3:** Data sources, study periods, age group of estimates used in the workbook method

| **Population size estimates** | | | **HBsAg prevalence estimates** | | | **anti-HCV and HCV-RNA prevalence estimates** | | |
| --- | --- | --- | --- | --- | --- | --- | --- | --- |
| **Age group (years)** | **Data Source** | **Period of Time** | **Age group (years)** | **Data Source** | **Period of Time** | **Age group (years)** | **Data Source** | **Period of Time** |
| **General Population excluding vulnerable groups** | | | | | | | | |
| < 18 | German Federal Statistic Office | 2013 | < 18 | RKI-Health Survey: KIGGS | 2003–2006 | - | - | - |
| ≥ 18 |  |  | 18–79 | RKI-Health Survey: DEGS1 | 2008–2011 | 18-79 | RKI-Health Survey: DEGS1 | 2008–2011 |
| **Migrants** | | | | | | | | |
| ≥ 18 | German Federal Statistic Office & Central Foreigners Register | 2013 | ≥ 18 | Schweitzer et al. 2015 | 1965–2013 | ≥ 18 | Gower et al. 2014 | 2010–2013 |
| **PWIO** | | | | | | | | |
| 15-64 | Kraus et al. 2019 & German Drug Report | 2017 & 2013 | 17–65 | DRUCK-Study | 2011–2014 | 17–65 | DRUCK-Study | 2011–2014 |
| **HIV+MSM** | | | | | | | | |
| 15-64 | RKI | 2013 | 18–64 | Krings et al. 2022 | 2013 | 18–64 | Krings et al. 2022 | 2013 |

HBsAg, Hepatitis B surface antigen; HBV, Hepatitis B virus; HCV, Hepatitis C virus; PWIO, People who inject opioids; HIV+MSM, HIV positive men who have sex with men; KIGGS, population-based Health Examination Survey for Children and Adolescents; DEGS1, population-based German Health Interview and Examination Survey for Adults; DRUCK, Infection and Behavior Survey among Injecting Drug Users (IVD) in Germany.
