# Supplementary material for: Estimated number of people infected with hepatitis B and C virus in Germany in 2013: a baseline prevalence estimate using the workbook method
Source: Front Public Health. 2025 Apr 7;13:1471256. doi: 10.3389/fpubh.2025.1471256 (PMC12009770; doi:10.3389/fpubh.2025.1471256)
Supplement: Supplementary file 4 [file Table_4.docx]

**Supplementary Table 4:**

Nationality specific population size, prevalence and number of HBV-infected adult migrants

| **Migrant population** | | **HBsAg prevalence estimate (%)** | | **Migrants with HBV** | |
| --- | --- | --- | --- | --- | --- |
| **Country of Nationality** | **Total number** | **Estimate** | **Low–High** | **Estimated number** | **Low–High** |
| **Afghanistan** | 43504 | 1.63 | 1.3–2.04 | 710 | 570–890 |
| **Albania** | 11769 | 7.79 | 7.56–8.03 | 920 | 890–950 |
| **Algier** | 11231 | 2.64 | 2.22–3.15 | 300 | 250–350 |
| **Angola** | 3086 | 12.74 | 11.37–14.26 | 390 | 350–440 |
| **Argentina** | 3920 | 0.77 | 0.77–0.78 | 30 | 30–30 |
| **Armenia** | 9388 | 0 | 0–0 | 0 | 0–0 |
| **Australia** | 9027 | 0.52 | 0.5–0.54 | 50 | 50–50 |
| **Austria** | 137650 | 2.5 | 1.07–5.72 | 3440 | 1470–7870 |
| **Azerbaijan** | 11847 | 2.77 | 1.71–4.45 | 330 | 200–530 |
| **Bangladesh** | 6478 | 3.09 | 2.99–3.21 | 200 | 190–210 |
| **Belarus** | 16041 | 4.6 | 4.2–5.02 | 740 | 670–810 |
| **Belgium** | 19389 | 0.7 | 0.48–1.02 | 140 | 90–200 |
| **Benin** | 1503 | 15.56 | 12.42–19.32 | 230 | 190–290 |
| **Bosnia and Herzegovina** | 116624 | 1.11 | 0.91–1.35 | 1290 | 1060–1570 |
| **Brasilia** | 30028 | 0.64 | 0.63–0.65 | 190 | 190–200 |
| **Bulgaria** | 122545 | 3.9 | 3.17–4.79 | 4780 | 3880–5870 |
| **Burkina Faso** | 1281 | 12.05 | 11.73–12.38 | 150 | 150–160 |
| **Cameroon** | 12408 | 10.03 | 9.32–10.78 | 1240 | 1160–1340 |
| **Canada** | 12562 | 0.66 | 0.53–0.82 | 80 | 70–100 |
| **Chile** | 5409 | 0.66 | 0.33–1.31 | 40 | 20–70 |
| **China** | 80573 | 5.41 | 5.4–5.43 | 4360 | 4350–4380 |
| **Colombia** | 10601 | 2.63 | 2.12–3.25 | 280 | 220–340 |
| **Cote d'Ivoire** | 2254 | 9.33 | 8.53–10.19 | 210 | 190–230 |
| **Croatia** | 173564 | 1.1 | 0.94–1.3 | 1910 | 1630–2260 |
| **Cuba** | 7201 | 1.3 | 0.63–2.66 | 90 | 50–190 |
| **Cyprus** | 1514 | 2.94 | 2.55–3.39 | 40 | 40–50 |
| **Czech Republic** | 40116 | 0.34 | 0.18–0.64 | 140 | 70–260 |
| **Denmark** | 16931 | 1.01 | 0.95–1.06 | 170 | 160–180 |
| **Dominican Republic** | 4792 | 4.1 | 2.67–6.24 | 200 | 130–300 |
| **Ecuador** | 3988 | 2 | 1.09–3.64 | 80 | 40–150 |
| **Egypt** | 12847 | 1.63 | 1.59–1.67 | 210 | 200–210 |
| **Eritrea** | 8511 | 2.49 | 2.32–2.68 | 210 | 200–230 |
| **Estonia** | 5027 | 0.58 | 0.42–0.74 | 30 | 20–40 |
| **Ethiopia** | 7680 | 4.65 | 4.38–4.93 | 360 | 340–380 |
| **Finland** | 11079 | 0 | 0–0 | 0 | 0–0 |
| **France** | 95692 | 0.25 | 0.24–0.26 | 240 | 230–250 |
| **Gambia** | 3465 | 8.46 | 7.52–9.5 | 290 | 260–330 |
| **Georgia** | 13429 | 2.07 | 1.53–2.81 | 280 | 210–380 |
| **Ghana** | 17240 | 12.96 | 12.48–13.45 | 2230 | 2150–2320 |
| **Greece** | 209301 | 0.67 | 0.65–0.69 | 1400 | 1360–1440 |
| **Guinea** | 3710 | 14.94 | 13.93–16 | 550 | 520–590 |
| **Guinea–Bissau** | 641 | 0 | 0–0 | 0 | 0–0 |
| **Hungary** | 119707 | 0.39 | 0.16–0.98 | 470 | 190–1170 |
| **Iceland** | 1285 | 0.14 | 0.04–0.51 | 0 | 0–10 |
| **India** | 51943 | 1.44 | 1.43–1.45 | 750 | 740–750 |
| **Indonesia** | 11869 | 1.83 | 1.73–1.93 | 220 | 210–230 |
| **Iran** | 45925 | 0.93 | 0.93–0.94 | 430 | 430–430 |
| **Iraq** | 51719 | 0.67 | 0.65–0.7 | 350 | 340–360 |
| **Ireland** | 9934 | 0.03 | 0.01–0.07 | 0 | 0–10 |
| **Israel** | 9298 | 0.83 | 0.8–0.86 | 80 | 70–80 |
| **Italy** | 355297 | 1.45 | 1.38–1.52 | 5150 | 4900–5400 |
| **Japan** | 24110 | 0.66 | 0.65–0.66 | 160 | 160–160 |
| **Jordan** | 6203 | 1.85 | 1.67–2.05 | 110 | 100–130 |
| **Kazakhstan** | 39638 | 3.81 | 2.14–6.68 | 1510 | 850–2650 |
| **Kenia** | 7777 | 5.31 | 4.91–5.75 | 410 | 380–450 |
| **Kosovo** | 69036 | 4.17 | 4.03–4.32 | 2880 | 2780–2980 |
| **Kuwait** | 508 | 0.8 | 0.65–0.97 | 0 | 0–0 |
| **Kyrgyzstan** | 6851 | 10.3 | 8.55–12.36 | 710 | 590–850 |
| **Latvia** | 22662 | 1.39 | 1.1–1.67 | 310 | 250–380 |
| **Lebanon** | 23728 | 1.07 | 0.95–1.2 | 250 | 230–280 |
| **Libya** | 5416 | 2.16 | 2.05–2.27 | 120 | 110–120 |
| **Lithuania** | 30534 | 1.7 | 1.55–1.86 | 520 | 470–570 |
| **Luxemburg** | 12356 | 0 | 0–0 | 0 | 0–0 |
| **Madagaskar** | 883 | 4.61 | 4.43–4.79 | 40 | 40–40 |
| **Malaysia** | 3725 | 0.71 | 0.67–0.74 | 30 | 20–30 |
| **Mali** | 1191 | 12.51 | 12.12–12.91 | 150 | 140–150 |
| **Malta** | 506 | 0 | 0–0 | 0 | 0–0 |
| **Mexico** | 9901 | 0.19 | 0.18–0.2 | 20 | 20–20 |
| **Moldova** | 9112 | 9.4 | 8.31–10.63 | 860 | 760–970 |
| **Mongolia** | 3089 | 9.07 | 8.41–9.78 | 280 | 260–300 |
| **Montenegro** | 7815 | 0 | 0–0 | 0 | 0–0 |
| **Morocco** | 48726 | 1.08 | 1.03–1.12 | 530 | 500–550 |
| **Nepal** | 3416 | 0.82 | 0.8–0.84 | 30 | 30–30 |
| **Netherlands** | 95010 | 0.38 | 0.37–0.39 | 360 | 350–370 |
| **New Zealand** | 2221 | 4.06 | 3.99–4.14 | 90 | 90–90 |
| **Nigeria** | 16286 | 9.81 | 9.63–9.99 | 1600 | 1570–1630 |
| **North Korea** | 1621 | 0 | 0–0 | 0 | 0–0 |
| **Norway** | 52850 | 0.01 | 0.01–0.02 | 10 | 10–10 |
| **Nothern Macedonia** | 5195 | 3.29 | 2.33–4.24 | 170 | 120–220 |
| **Pakistan** | 31427 | 2.75 | 2.72–2.78 | 860 | 850–870 |
| **Palestine** | 1446 | 1.8 | 1.08–3 | 30 | 20–40 |
| **Peru** | 7706 | 1.74 | 1.47–2.07 | 130 | 110–160 |
| **Philippines** | 22973 | 4.18 | 4.08–4.28 | 960 | 940–980 |
| **Poland** | 510522 | 1.01 | 0.77–1.32 | 5160 | 3930–6740 |
| **Portugal** | 91384 | 0.94 | 0.69–1.28 | 860 | 630–1170 |
| **Romania** | 224249 | 5.66 | 5.54–5.78 | 12690 | 12420–12960 |
| **Russia** | 170567 | 3.13 | 3.01–3.25 | 5340 | 5130–5540 |
| **Saudia Arabia** | 2113 | 2.85 | 2.79–2.91 | 60 | 60–60 |
| **Schweden** | 14561 | 0.77 | 0.62–0.96 | 110 | 90–140 |
| **Senegal** | 2534 | 11.78 | 10.96–12.65 | 300 | 280–320 |
| **Serbia** | 104324 | 0.48 | 0.43–0.55 | 500 | 450–570 |
| **Sierra Leone** | 1875 | 8.35 | 5.94–11.63 | 160 | 110–220 |
| **Singapore** | 1485 | 2.97 | 2.75–3.22 | 40 | 40–50 |
| **Slovakia** | 35064 | 1.74 | 1.64–1.85 | 610 | 580–650 |
| **Slovenia** | 19076 | 0.28 | 0.25–0.3 | 50 | 50–60 |
| **Somalia** | 7255 | 19.32 | 17.13–21.72 | 1400 | 1240–1580 |
| **South Africa** | 3957 | 6.56 | 5.85–7.34 | 260 | 230–290 |
| **South Korea** | 18469 | 4.36 | 4.36–4.37 | 810 | 810–810 |
| **Spain** | 99231 | 0.3 | 0.28–0.32 | 300 | 280–320 |
| **Sri Lanka** | 19383 | 2.5 | 1.89–3.3 | 480 | 370–640 |
| **Sudan** | 2252 | 22.33 | 20.06–24.78 | 500 | 450–560 |
| **Switzerland** | 30443 | 0.18 | 0.1–0.32 | 50 | 30–100 |
| **Syria** | 38157 | 2.62 | 2.17–3.16 | 1000 | 830–1210 |
| **Taiwan** | 5207 | 0 | 0–0 | 0 | 0–0 |
| **Tajikistan** | 937 | 7.18 | 5.5–9.32 | 70 | 50–90 |
| **Thailand** | 50459 | 6.12 | 6.07–6.18 | 3090 | 3060–3120 |
| **Togo** | 6547 | 10.93 | 7.53–15.63 | 720 | 490–1020 |
| **Tunesia** | 20390 | 6.04 | 5.82–6.28 | 1230 | 1190–1280 |
| **Türkiye** | 954558 | 4 | 3.99–4.02 | 38180 | 38090–38370 |
| **Ukraine** | 100553 | 1.45 | 1.11–1.89 | 1460 | 1120–1900 |
| **United Arab Emirates** | 912 | 0.68 | 0.4–1.17 | 10 | 0–10 |
| **United Kingdom** | 82405 | 0.01 | 0–0.01 | 10 | 0–10 |
| **United States of America** | 85315 | 0.26 | 0.23–0.29 | 220 | 200–250 |
| **Uzbekistan** | 7179 | 11.67 | 10.05–13.52 | 840 | 720–970 |
| **Venezuela** | 3477 | 0.4 | 0.37–0.43 | 10 | 10–10 |
| **Vietnam** | 57779 | 10.76 | 10.25–11.28 | 6220 | 5920–6520 |
| **Yemen** | 2048 | 8.19 | 7.76–8.63 | 170 | 160–180 |

HBsAg, Hepatitis B surface antigen; HBV, Hepatitis B virus
