# Supplementary material for: Estimated number of people infected with hepatitis B and C virus in Germany in 2013: a baseline prevalence estimate using the workbook method
Source: Front Public Health. 2025 Apr 7;13:1471256. doi: 10.3389/fpubh.2025.1471256 (PMC12009770; doi:10.3389/fpubh.2025.1471256)
Supplement: Supplementary file 5 [file Table_5.docx]

**Supplementary Table 5:**

Nationality specific population size, prevalence and number of HCV-infected and ever–HCV–infected adult migrants

| **Migrant population** | | **anti-HCV prevalence estimate (%)** | | **Migrants**  **ever-HCV infected** | | **Migrants with HCV–infection** | |
| --- | --- | --- | --- | --- | --- | --- | --- |
| **Country of Nationality** | **Total number** | **Estimate** | **Low–High** | **Estimated number** | **Low–High** | **Estimated number** | **Low–High** |
| **Afghanistan** | 43504 | 1.1 | 0.6–1.9 | 480 | 260–830 | 350 | 190–640 |
| **Albania** | 11769 | 0 | 0–0 | 0 | 0–0 | 0 | 0–0 |
| **Algier** | 11231 | 1.4 | 0.2–2.5 | 160 | 20–280 | 120 | 20–220 |
| **Angola** | 3086 | 0 | 0–0 | 0 | 0–0 | 0 | 0–0 |
| **Argentina** | 3920 | 1.5 | 0.5–2.5 | 60 | 20–100 | 40 | 10–80 |
| **Armenia** | 9388 | 0 | 0–0 | 0 | 0–0 | 0 | 0–0 |
| **Australia** | 9027 | 1.7 | 1.2–1.8 | 150 | 110–160 | 110 | 80–130 |
| **Austria** | 137650 | 0.5 | 0.1–0.7 | 690 | 140–960 | 510 | 100–750 |
| **Azerbaijan** | 11847 | 3.1 | 1–6.7 | 370 | 120–790 | 270 | 80–620 |
| **Bangladesh** | 6478 | 1.3 | 0.2–2.2 | 80 | 10–140 | 60 | 10–110 |
| **Belarus** | 16041 | 1.3 | 0.9–2.9 | 210 | 140–470 | 150 | 100–360 |
| **Belgium** | 19389 | 0.9 | 0.1–1.2 | 170 | 20–230 | 130 | 10–180 |
| **Benin** | 1503 | 3.6 | 3.6–12.8 | 50 | 50–190 | 40 | 40–150 |
| **Bosnia and Herzegovina** | 116624 | 0 | 0–0 | 0 | 0–0 | 0 | 0–0 |
| **Brasilia** | 30028 | 1.58 | 1.12–1.64 | 470 | 340–490 | 350 | 240–380 |
| **Bulgaria** | 122545 | 1.1 | 0.3–2.4 | 1350 | 370–2940 | 1000 | 260–2290 |
| **Burkina Faso** | 1281 | 0 | 0–0 | 0 | 0–0 | 0 | 0–0 |
| **Cameroon** | 12408 | 11.6 | 4.3–29.7 | 1440 | 530–3690 | 1070 | 380–2870 |
| **Canada** | 12562 | 1.1 | 0.6–1.3 | 140 | 80–160 | 100 | 50–130 |
| **Chile** | 5409 | 0 | 0–0 | 0 | 0–0 | 0 | 0–0 |
| **China** | 80573 | 1.3 | 0.4–2 | 1050 | 320–1610 | 780 | 230–1260 |
| **Colombia** | 10601 | 0 | 0–0 | 0 | 0–0 | 0 | 0–0 |
| **Cote d'Ivoire** | 2254 | 3.3 | 0.8–12.8 | 70 | 20–290 | 60 | 10–230 |
| **Croatia** | 173564 | 0 | 0–0 | 0 | 0–0 | 0 | 0–0 |
| **Cuba** | 7201 | 0 | 0–0 | 0 | 0–0 | 0 | 0–0 |
| **Cyprus** | 1514 | 0.6 | 0.5–1.9 | 10 | 10–30 | 10 | 10–20 |
| **Czech Republic** | 40116 | 0.66 | 0.2–0.7 | 260 | 80–280 | 200 | 60–220 |
| **Denmark** | 16931 | 0.7 | 0.5–0.7 | 120 | 80–120 | 90 | 60–90 |
| **Dominican Republic** | 4792 | 0 | 0–0 | 0 | 0–0 | 0 | 0–0 |
| **Ecuador** | 3988 | 0 | 0–0 | 0 | 0–0 | 0 | 0–0 |
| **Egypt** | 12847 | 14.7 | 10.3–18 | 1890 | 1320–2310 | 1400 | 940–1800 |
| **Eritrea** | 8511 | 0 | 0–0 | 0 | 0–0 | 0 | 0–0 |
| **Estonia** | 5027 | 0 | 0–0 | 0 | 0–0 | 0 | 0–0 |
| **Ethiopia** | 7680 | 1.3 | 0.7–5.8 | 100 | 50–450 | 70 | 40–350 |
| **Finland** | 11079 | 0.7 | 0.6–0.9 | 80 | 70–100 | 60 | 50–80 |
| **France** | 95692 | 0.6 | 0.4–1.1 | 570 | 380–1050 | 420 | 270–820 |
| **Gambia** | 3465 | 2.1 | 1.4–2.9 | 70 | 50–100 | 50 | 30–80 |
| **Georgia** | 13429 | 6.7 | 5.6–7.3 | 900 | 750–980 | 670 | 530–760 |
| **Ghana** | 17240 | 0 | 0–0 | 0 | 0–0 | 0 | 0–0 |
| **Greece** | 209301 | 1.9 | 0.5–2.6 | 3980 | 1050–5440 | 2940 | 740–4240 |
| **Guinea** | 3710 | 0 | 0–0 | 0 | 0–0 | 0 | 0–0 |
| **Guinea–Bissau** | 641 | 0 | 0–0 | 0 | 0–0 | 0 | 0–0 |
| **Hungary** | 119707 | 0.8 | 0.4–2.7 | 960 | 480–3230 | 710 | 340–2520 |
| **Iceland** | 1285 | 0 | 0–0 | 0 | 0–0 | 0 | 0–0 |
| **India** | 51943 | 0.8 | 0.4–1 | 420 | 210–520 | 310 | 150–410 |
| **Indonesia** | 11869 | 0.8 | 0.4–2 | 90 | 50–240 | 70 | 30–190 |
| **Iran** | 45925 | 0.5 | 0.2–1 | 230 | 90–460 | 170 | 70–360 |
| **Iraq** | 51719 | 3.2 | 0.3–3.2 | 1650 | 160–1650 | 1220 | 110–1290 |
| **Ireland** | 9934 | 1.1 | 0.7–1.6 | 110 | 70–160 | 80 | 50–120 |
| **Israel** | 9298 | 2 | 0.9–2 | 190 | 80–190 | 140 | 60–150 |
| **Italy** | 355297 | 2 | 1.6–7.3 | 7110 | 5680–25940 | 5260 | 4040–20230 |
| **Japan** | 24110 | 1.5 | 0.5–2.2 | 360 | 120–530 | 270 | 90–410 |
| **Jordan** | 6203 | 0 | 0–0 | 0 | 0–0 | 0 | 0–0 |
| **Kazakhstan** | 39638 | 3.3 | 1–6.7 | 1310 | 400–2660 | 970 | 280–2070 |
| **Kenia** | 7777 | 0 | 0–0 | 0 | 0–0 | 0 | 0–0 |
| **Kosovo** | 69036 | 0 | 0–0 | 0 | 0–0 | 0 | 0–0 |
| **Kuwait** | 508 | 0 | 0–0 | 0 | 0–0 | 0 | 0–0 |
| **Kyrgyzstan** | 6851 | 2.5 | 1.6–6.7 | 170 | 110–460 | 130 | 80–360 |
| **Latvia** | 22662 | 2.4 | 1.7–3.3 | 540 | 390–750 | 400 | 270–580 |
| **Lebanon** | 23728 | 0 | 0–0 | 0 | 0–0 | 0 | 0–0 |
| **Libya** | 5416 | 1.2 | 1.2–2.3 | 60 | 60–120 | 50 | 50–100 |
| **Lithuania** | 30534 | 2.9 | 0.7–3 | 890 | 210–920 | 660 | 150–710 |
| **Luxemburg** | 12356 | 0.91 | 0.56–0.93 | 110 | 70–110 | 80 | 50–90 |
| **Madagaskar** | 883 | 1.2 | 0.8–1.7 | 10 | 10–20 | 10 | 10–10 |
| **Malaysia** | 3725 | 1.5 | 0.3–7.7 | 60 | 10–290 | 40 | 10–220 |
| **Mali** | 1191 | 0 | 0–0 | 0 | 0–0 | 0 | 0–0 |
| **Malta** | 506 | 0 | 0–0 | 0 | 0–0 | 0 | 0–0 |
| **Mexico** | 9901 | 1.4 | 1.1–1.6 | 140 | 110–160 | 100 | 80–120 |
| **Moldova** | 9112 | 4.5 | 2.3–4.5 | 410 | 210–410 | 300 | 150–320 |
| **Mongolia** | 3089 | 10.8 | 8.7–15.6 | 330 | 270–480 | 250 | 190–380 |
| **Montenegro** | 7815 | 0 | 0–0 | 0 | 0–0 | 0 | 0–0 |
| **Morocco** | 48726 | 0 | 0–0 | 0 | 0–0 | 0 | 0–0 |
| **Nepal** | 3416 | 0 | 0–0 | 0 | 0–0 | 0 | 0–0 |
| **Netherlands** | 95010 | 0.2 | 0.1–0.4 | 190 | 100–380 | 140 | 70–300 |
| **New Zealand** | 2221 | 1.9 | 0.8–2.2 | 40 | 20–50 | 30 | 10–40 |
| **Nigeria** | 16286 | 8.4 | 3.9–12.8 | 1370 | 640–2080 | 1010 | 450–1630 |
| **North Korea** | 1621 | 0 | 0–0 | 0 | 0–0 | 0 | 0–0 |
| **Norway** | 52850 | 0.7 | 0.6–0.9 | 370 | 320–480 | 270 | 230–370 |
| **Nothern Macedonia** | 5195 | 0 | 0–0 | 0 | 0–0 | 0 | 0–0 |
| **Pakistan** | 31427 | 6.7 | 1.6–10 | 2110 | 500–3140 | 1560 | 360–2450 |
| **Palestine** | 1446 | 0 | 0–0 | 0 | 0–0 | 0 | 0–0 |
| **Peru** | 7706 | 1.2 | 0.4–1.6 | 90 | 30–120 | 70 | 20–100 |
| **Philippines** | 22973 | 0.9 | 0.3–2 | 210 | 70–460 | 150 | 50–360 |
| **Poland** | 510522 | 0.9 | 0.6–1.1 | 4590 | 3060–5620 | 3400 | 2170–4380 |
| **Portugal** | 91384 | 1.8 | 0.5–2.9 | 1640 | 460–2650 | 1220 | 320–2070 |
| **Romania** | 224249 | 3.2 | 2.9–3.6 | 7180 | 6500–8070 | 5310 | 4620–6300 |
| **Russia** | 170567 | 4.1 | 1.2–5.6 | 6990 | 2050–9550 | 5170 | 1450–7450 |
| **Saudia Arabia** | 2113 | 1.5 | 0.6–7.3 | 30 | 10–150 | 20 | 10–120 |
| **Schweden** | 14561 | 0.7 | 0.5–0.7 | 100 | 70–100 | 80 | 50–80 |
| **Senegal** | 2534 | 0 | 0–0 | 0 | 0–0 | 0 | 0–0 |
| **Serbia** | 104324 | 0 | 0–0 | 0 | 0–0 | 0 | 0–0 |
| **Sierra Leone** | 1875 | 0 | 0–0 | 0 | 0–0 | 0 | 0–0 |
| **Singapore** | 1485 | 0 | 0–0 | 0 | 0–0 | 0 | 0–0 |
| **Slovakia** | 35064 | 1.4 | 0.9–2 | 490 | 320–700 | 360 | 220–550 |
| **Slovenia** | 19076 | 0 | 0–0 | 0 | 0–0 | 0 | 0–0 |
| **Somalia** | 7255 | 0 | 0–0 | 0 | 0–0 | 0 | 0–0 |
| **South Africa** | 3957 | 1.7 | 1–2.5 | 70 | 40–100 | 50 | 30–80 |
| **South Korea** | 18469 | 0.8 | 0.2–2.1 | 150 | 40–390 | 110 | 30–300 |
| **Spain** | 99231 | 1.7 | 0.4–2.6 | 1690 | 400–2580 | 1250 | 280–2010 |
| **Sri Lanka** | 19383 | 0 | 0–0 | 0 | 0–0 | 0 | 0–0 |
| **Sudan** | 2252 | 0 | 0–0 | 0 | 0–0 | 0 | 0–0 |
| **Switzerland** | 30443 | 1.5 | 0.7–1.8 | 460 | 210–550 | 340 | 150–430 |
| **Syria** | 38157 | 0 | 0–0 | 0 | 0–0 | 0 | 0–0 |
| **Taiwan** | 5207 | 4.4 | 2.5–6.3 | 230 | 130–330 | 170 | 90–260 |
| **Tajikistan** | 937 | 3.1 | 1.1–6.7 | 30 | 10–60 | 20 | 10–50 |
| **Thailand** | 50459 | 2.7 | 1.8–3.7 | 1360 | 910–1870 | 1010 | 640–1460 |
| **Togo** | 6547 | 0 | 0–0 | 0 | 0–0 | 0 | 0–0 |
| **Tunesia** | 20390 | 1.3 | 0.3–2.5 | 270 | 60–510 | 200 | 40–400 |
| **Türkiye** | 954558 | 1 | 0.6–2.1 | 9550 | 5730–20050 | 7060 | 4070–15640 |
| **Ukraine** | 100553 | 3.6 | 0.9–4.5 | 3620 | 900–4520 | 2680 | 640–3530 |
| **United Arab Emirates** | 912 | 0 | 0–0 | 0 | 0–0 | 0 | 0–0 |
| **United Kingdom** | 82405 | 0.6 | 0.4–1.2 | 490 | 330–990 | 370 | 230–770 |
| **United States of America** | 85315 | 1.3 | 1.2–2.4 | 1110 | 1020–2050 | 820 | 730–1600 |
| **Uzbekistan** | 7179 | 11.3 | 6.4–13.1 | 810 | 460–940 | 600 | 330–730 |
| **Venezuela** | 3477 | 1.5 | 0.3–2.6 | 50 | 10–90 | 40 | 10–70 |
| **Vietnam** | 57779 | 0 | 0–0 | 0 | 0–0 | 0 | 0–0 |
| **Yemen** | 2048 | 2.2 | 1.1–3.5 | 50 | 20–70 | 30 | 20–60 |

HCV, Hepatitis C virus
