# Supplementary material for: Estimated number of people infected with hepatitis B and C virus in Germany in 2013: a baseline prevalence estimate using the workbook method
Source: Front Public Health. 2025 Apr 7;13:1471256. doi: 10.3389/fpubh.2025.1471256 (PMC12009770; doi:10.3389/fpubh.2025.1471256)
Supplement: Supplementary file 6 [file Table_6.docx]

**Supplementary Table 6**:

Population size and number of HBV-infected migrants with regional prevalence estimates

| **Migrant population** | | **HBsAg prevalence estimate (%)** | | | **Migrants with HBV** | |
| --- | --- | --- | --- | --- | --- | --- |
| **Country of Nationality** | **Total number** | **Region of estimate*** | **Estimate*** | **Low-High*** | **Estimated number** | **Low-High** |
| **Finland** | 11079 | Northern/Western Europe | 0.55 | 0.34–0.71 | 61 | 40–80 |
| **Guinea-Bissau** | 641 | Western Africa | 13.17 | 10.18–16.17 | 84 | 70–100 |
| **North Korea** | 1621 | Eastern Asia | 8.97 | 8.47–9.48 | 145 | 140–150 |
| **Luxemburg** | 12356 | Northern/Western Europe | 0.55 | 0.34–0.71 | 68 | 40–90 |
| **Malta** | 506 | Southern Europe | 2.13 | 1.71–2.54 | 11 | 10–10 |
| **Montenegro** | 7815 | Eastern Europe | 3.29 | 2.33–4.24 | 257 | 180–330 |
| **Taiwan** | 5207 | South East Asia | 9.23 | 8.15–10.48 | 481 | 420–550 |
| **Total** | **39224** | **-** | **-** | **-** | **1107** | **900–1310** |

HBsAg, Hepatitis B surface antigen; HBV, Hepatitis B virus

*taken from Kowdley et al.
