# Supplementary material for: Estimated number of people infected with hepatitis B and C virus in Germany in 2013: a baseline prevalence estimate using the workbook method
Source: Front Public Health. 2025 Apr 7;13:1471256. doi: 10.3389/fpubh.2025.1471256 (PMC12009770; doi:10.3389/fpubh.2025.1471256)
Supplement: Supplementary file 7 [file Table_7.docx]

**Supplementary Table 7**: Population size and number of HCV-infected and ever–HCV-infected migrants with regional prevalence estimates

| **Migrant population** | | **Region of prevalence estimate*** | **anti-HCV prevalence estimate (%) *** | | **viremic HCV prevalence estimate (%) *** | | | **Migrants**  **ever-HCV infected** | | **Migrants with HCV** | |
| --- | --- | --- | --- | --- | --- | --- | --- | --- | --- | --- | --- |
| **Country of Nationality** | **Total number** |  | **Estimate** | **Low–High** | **Estimate** | | **Low–High** | **Estimated number** | **Low–High** | **Estimated number** | **Low–High** |
| Albania | 11769 | Central Europe | 1.3 | 1.1–1.6 | 1 | | 0.9–1.2 | 150 | 130–190 | 120 | 110–140 |
| Angola | 3086 | Sub-Saharan Central Africa | 4.2 | 2.4–9.2 | 2.6 | | 1.5–5.5 | 130 | 70–280 | 80 | 50–170 |
| Armenia | 9388 | Central Asia | 5.4 | 3.5–6.8 | 2.3 | | 1.5–3 | 510 | 330–640 | 220 | 140–280 |
| Bosnia and Herzegovina | 116624 | Central Europe | 1.3 | 1.1–1.6 | 1 | 0.9–1.2 | | 1520 | 1280–1870 | 1170 | 1050–1400 |
| Burkina Faso | 1281 | Sub-Saharan West Africa | 5.3 | 2.9–9.1 | 4.1 | | 2.3–6.7 | 70 | 40–120 | 50 | 30–90 |
| Chile | 5409 | Latin America, Andean | 0.9 | 0.4–1.3 | 0.6 | | 0.3–0.9 | 50 | 20–70 | 30 | 20–50 |
| Colombia | 10601 | Latin America, Central | 1 | 0.9–1.2 | 0.8 | | 0.6–1.1 | 110 | 100–130 | 80 | 60–120 |
| Croatia | 173564 | Central Europe | 1.3 | 1.1–1.6 | 1 | | 0.9–1.2 | 2260 | 1910–2780 | 1740 | 1560–2080 |
| Cuba | 7201 | Caribbean | 0.8 | 0.2–1.3 | 0.6 | | 0.1–0.9 | 60 | 10–90 | 40 | 10–60 |
| Dominican Republic | 4792 | Caribbean | 0.8 | 0.2–1.3 | 0.6 | | 0.1–0.9 | 40 | 10–60 | 30 | 0–40 |
| Ecuador | 3988 | Latin America, Andean | 0.9 | 0.4–1.3 | 0.6 | | 0.3–0.9 | 40 | 20–50 | 20 | 10–40 |
| Eritrea | 8511 | Middle East/North Africa | 3.1 | 2.5–3.9 | 2.1 | | 1.7–2.6 | 260 | 210–330 | 180 | 140–220 |
| Estonia | 5027 | Eastern Europe | 3.3 | 1.6–4.5 | 2.3 | | 1.1–3 | 170 | 80–230 | 120 | 60–150 |
| Ghana | 17240 | Sub-Saharan West Africa | 5.3 | 2.9–9.1 | 4.1 | | 2.3–6.7 | 910 | 500–1570 | 710 | 400–1160 |
| Guinea | 3710 | Sub-Saharan West Africa | 5.3 | 2.9–9.1 | 4.1 | | 2.3–6.7 | 200 | 110–340 | 150 | 90–250 |
| Guinea–Bissau | 641 | Sub-Saharan West Africa | 5.3 | 2.9–9.1 | 4.1 | | 2.3–6.7 | 30 | 20–60 | 30 | 10–40 |
| Iceland | 1285 | Western Europe | 0.9 | 0.7–1.5 | 0.6 | | 0.5–1 | 10 | 10–20 | 10 | 10–10 |
| Jordan | 6203 | Middle East/North Africa | 3.1 | 2.5–3.9 | 2.1 | | 1.7–2.6 | 190 | 160–240 | 130 | 110–160 |
| Kenia | 7777 | Sub-Saharan East Africa | 1 | 0.6–3.1 | 0.6 | | 0.4–2 | 80 | 50–240 | 50 | 30–160 |
| Kosovo | 69036 | Central Europe | 1.3 | 1.1–1.6 | 1 | | 0.9–1.2 | 900 | 760–1100 | 690 | 620–830 |
| Kuwait | 508 | Middle East/North Africa | 3.1 | 2.5–3.9 | 2.1 | | 1.7–2.6 | 20 | 10–20 | 10 | 10–10 |
| Lebanon | 23728 | Middle East/North Africa | 3.1 | 2.5–3.9 | 2.1 | | 1.7–2.6 | 740 | 590–930 | 500 | 400–620 |
| Mali | 1191 | Sub-Saharan West Africa | 5.3 | 2.9–9.1 | 4.1 | | 2.3–6.7 | 60 | 30–110 | 50 | 30–80 |
| Malta | 506 | Western Europe | 0.9 | 0.7–1.5 | 0.6 | | 0.5–1 | 0 | 0–10 | 0 | 0–10 |
| Montenegro | 7815 | Central Europe | 1.3 | 1.1–1.6 | 1 | | 0.9–1.2 | 100 | 90–130 | 80 | 70–90 |
| Morocco | 48726 | Middle East/North Africa | 3.1 | 2.5–3.9 | 2.1 | | 1.7–2.6 | 1510 | 1220–1900 | 1020 | 830–1270 |
| Nepal | 3416 | South Asia | 1.1 | 0.7–1.5 | 0.9 | | 0.5–1.2 | 40 | 20–50 | 30 | 20–40 |
| North Korea | 1621 | Central Asia | 5.4 | 3.5–6.8 | 2.3 | | 1.5–3 | 90 | 60–110 | 40 | 20–50 |
| Nothern Macedonia | 5195 | Central Europe | 1.3 | 1.1–1.6 | 1 | | 0.9–1.2 | 70 | 60–80 | 50 | 50–60 |
| Palestine | 1446 | Middle East/North Africa | 3.1 | 2.5–3.9 | 2.1 | | 1.7–2.6 | 40 | 40–60 | 30 | 20–40 |
| Senegal | 2534 | Sub-Saharan West Africa | 5.3 | 2.9–9.1 | 4.1 | | 2.3–6.7 | 130 | 70–230 | 100 | 60–170 |
| Serbia | 104324 | Central Europe | 1.3 | 1.1–1.6 | 1 | | 0.9–1.2 | 1360 | 1150–1670 | 1040 | 940–1250 |
| Sierra Leone | 1875 | Sub-Saharan West Africa | 5.3 | 2.9–9.1 | 4.1 | | 2.3–6.7 | 100 | 50–170 | 80 | 40–130 |
| Singapore | 1485 | South Asia | 1.1 | 0.7–1.5 | 0.9 | | 0.5–1.2 | 20 | 10–20 | 10 | 10–20 |
| Slovenia | 19076 | Central Europe | 1.3 | 1.1–1.6 | 1 | | 0.9–1.2 | 250 | 210–310 | 190 | 170–230 |
| Somalia | 7255 | Middle East/North Africa | 3.1 | 2.5–3.9 | 2.1 | | 1.7–2.6 | 220 | 180–280 | 150 | 120–190 |
| Sri Lanka | 19383 | Southeast Asia | 1 | 0.8–1.8 | 0.7 | | 0.5–1.1 | 190 | 160–350 | 140 | 100–210 |
| Sudan | 2252 | Middle East/North Africa | 3.1 | 2.5–3.9 | 2.1 | | 1.7–2.6 | 70 | 60–90 | 50 | 40–60 |
| Syria | 38157 | Middle East/North Africa | 3.1 | 2.5–3.9 | 2.1 | | 1.7–2.6 | 1180 | 950–1490 | 800 | 650–990 |
| Togo | 6547 | Sub-Saharan West Africa | 5.3 | 2.9–9.1 | 4.1 | | 2.3–6.7 | 350 | 190–600 | 270 | 150–440 |
| United Arab Emirates | 912 | Middle East/North Africa | 3.1 | 2.5–3.9 | 2.1 | | 1.7–2.6 | 30 | 20–40 | 20 | 20–20 |
| Vietnam | 57779 | Southeast Asia | 1 | 0.8–1.8 | 0.7 | | 0.5–1.1 | 580 | 460–1040 | 400 | 290–640 |

HCV, Hepatitis C virus

***(**by Gower et al.
